# Ligand-dependent responses of the silkworm prothoracicotropic hormone receptor, Torso, are maintained by unusual intermolecular disulfide bridges in the transmembrane region

**DOI:** 10.1038/srep22437

**Published:** 2016-03-01

**Authors:** Tadafumi Konogami, Yiwen Yang, Mari H. Ogihara, Juri Hikiba, Hiroshi Kataoka, Kazuki Saito

**Affiliations:** 1 Department of Integrated Biosciences, Graduate School of Frontier Sciences, The University of Tokyo, Kashiwa, Chiba 277-8562, Japan

## Abstract

The insect membrane-protein, Torso, is a member of the receptor-tyrosine-kinase family, and is activated by its ligand, prothoracicotropic hormone (PTTH). Although PTTH is one of the most important regulators of insect development, the mechanism of Torso activation by the hormone has remained elusive. In this study, using heterologous expression in cultured *Drosophila* S2 cells, we detected ligand-independent dimerization of silkworm Torso, and found that the receptor molecules in the dimer were linked by intermolecular disulfide bridges. By examining the oligomerization states of several truncation and substitution mutants of Torso, atypical cysteine residues in the transmembrane region were identified as being responsible for the intermolecular linkage in the dimer. The replacement of all of the cysteines in the region with phenylalanines abolished the disulfide-bond-mediated dimerization; however, non-covalent dimerization of the mutant was detected using a cross-linking reagent, both with and without ligand stimulation. This non-covalent dimerization caused apparent receptor autophosphorylation independently of the ligand stimulation, but did not promote the ERK phosphorylation in the downstream signaling pathway. The unique Torso structure with the intermolecular disulfide bridges in the transmembrane region is necessary to maintain the ligand-dependent receptor functions of autophosphorylation and downstream activation.

Cellular-membrane receptors play important roles as mediators, in the signal transduction of extracellular stimuli to the activation of intracellular biochemical pathways. The receptors are composed of several types of membrane proteins, such as G-protein-coupled receptors (GPCRs), enzyme-linked receptors, ion-channel-linked receptors, and others^1^. To perform their transduction activities, GPCRs usually require other protein partners such as G-proteins, while the enzyme-linked receptors can activate the intracellular pathways by themselves, using the catalytic domains in their own intracellular regions. For example, receptor tyrosine kinases (RTKs) commonly have intracellular tyrosine kinase domains, which phosphorylate the tyrosine residues of the receptors themselves and those within other intracellular protein molecules.

Insects also utilize cellular-membrane RTKs to receive extracellular hormonal signals. Among the insect hormonal receptors, Torso, a member of the RTK family, was recently reported to contribute to the activation of prothoracic gland cells in response to stimulation by prothoracicotropic hormone (PTTH)^2^, although Torso was originally discovered as an anteroposterior axis determinant in *Drosophila* embryos^3^,^4^,^5^. In *Drosophila* larvae, Torso is expressed specifically in the prothoracic glands, and the *torso* knock-down caused delayed larval development^2^. This phenotype of the knocked-down larvae may be derived from the lack of the molting hormone, ecdysone, since PTTH promotes ecdysone biosynthesis in the prothoracic glands through the stimulation of its receptor Torso, on the surface of the gland cells. Silkworm Torso was also activated by its ligand, silkworm PTTH, when it was transiently expressed in cultured *Drosophila* S2 cells. In response to the PTTH stimulation, the phosphorylation of extracellular signal-regulated kinase (ERK) in the Torso-expressing cells was robustly promoted^2^. Enhanced ERK phosphorylation is often observed in RTK-expressing cells, in response to stimulation by their corresponding ligands.

Although PTTH plays very important roles in insect development^6^, the detailed activation mechanism of its receptor Torso by the hormone has remained unelucidated. In response to ligand stimulation, an RTK generally induces the phosphorylation of tyrosine residues in its own intracellular region, but the process leading to the receptor autophosphorylation varies among the different RTK subclasses. The RTKs are classified into three major groups, subclasses I–III, according to the extracellular domain structures^7^,^8^, while they share similar tyrosine kinase domains in their intracellular regions. The members of the subclass-I RTKs have two extracellular cysteine-rich domains in a single polypeptide chain, which passes through the cellular membrane. Epidermal growth factor receptor (EGFR), a typical member of the subclass-I RTKs, adopts a non-covalently-associated dimeric form on the cellular membrane in response to ligand stimulation, and the kinase domains of the two receptor molecules in the dimer consequently phosphorylate the intracellular tyrosine residues of the partner^9^.

The members of the subclass-II RTKs have one cysteine-rich domain in an extracellular α chain, and the α chain is linked to another membrane-spanning β chain by a disulfide bond. Two sets of the αβ heterodimers are further linked to each other by additional disulfide bridges between the α chains, resulting in the formation of an α_2_β_2_ heterotetramer^10^. Insulin receptor is the most thoroughly characterized member of the subclass-II RTKs. The receptor adopts the α_2_β_2_ tetrameric form before stimulation, and does not form a higher oligomer even after ligand stimulation. The autophosphorylation of the subclass-II RTKs may be driven by ligand-induced conformational changes.

The insect RTK Torso resembles the members of subclass III, in terms of the extracellular domain composition. They are commonly composed of a single polypeptide chain, and their extracellular regions lack a cysteine-rich domain, while the cysteine distribution in the other regions is mostly conserved. The autophosphorylation of the subclass-III RTKs is always accompanied by receptor dimerization, as in the subclass-I RTKs, but it remains to be determined whether Torso shares a common activation mechanism with the receptors of subclasses I and III, since Torso has a very different extracellular cysteine distribution from those common among the subclass-III RTKs.

Torso has recently attracted attention as a key molecule in PTTH-driven insect development, but biochemical studies of its activation mechanism have lagged behind. One of the reasons is the poor availability of the recombinant ligand. The production of recombinant PTTH was attempted many times by heterologous expression using *Escherichia coli*, but the yields were very low, because burdensome refolding procedures were always required to obtain the active ligand^11^. Recently, however, we established an efficient method for preparing silkworm PTTH, using the *Brevibacillus* (formally known as *Bacillus brevis*) secretory expression system^12^,^13^. In this study, to elucidate the initial step of the ligand-induced Torso activation process, we examined the oligomerization of silkworm Torso triggered by recombinant silkworm PTTH, by employing the heterologous expression of FLAG-tagged receptors in cultured *Drosophila* S2 cells. We discovered a novel disulfide-bond-mediated dimer structure of Torso in the course of our receptor-oligomerization analyses, and now report the locations and the possible role of the intermolecular disulfide bridges.

## Results

### Uniqueness of the Torso extracellular region

Like the members of the subclass-III RTKs, Torso lacks a cysteine-rich domain in its extracellular region, but the primary structure of the Torso extracellular region shares only low similarity with those of the subclass-III RTKs (Fig. S1 in the Supplementary Information). Insects also have a variety of RTKs to receive hormonal signals, and several insect RTKs have been classified into subclass III. For example, the protein products of the *Drosophila pvr* (PDGF- and VEGF-receptor related), *btl* (breathless), and *htl* (heartless) genes are receptors that are the fruit-fly homologues of human subclass-III RTKs, such as platelet-derived growth factor receptor (PDGFR), vascular endothelial growth factor receptor (VEGFR), and fibroblast growth factor receptor (FGFR)^14^,^15^,^16^. However, Torso is quite different from these subclass-III RTK members, in terms of its domain structure and cysteine distribution in the extracellular region. Further information about the uniqueness of the Torso extracellular region is provided in the Supplementary Information.

### Oligomerization of silkworm Torso in cultured *Drosophila* S2 cells

Even though the extracellular region of Torso has a unique structure, the receptor actually facilitated the phosphorylation of intracellular ERK in response to ligand stimulation^2^, in a similar manner to most other RTKs. In this study, to elucidate the activation mechanism of Torso, oligomerization of the receptor was examined, because this is the initial step for the ligand-dependent activation processes of many single-polypeptide RTKs. Since silkworm PTTH was the only available recombinant ligand, the receptor from the same insect species, silkworm Torso, was expressed heterologously in cultured *Drosophila* S2 cells. This cell culture system is one of the most simple and convenient ways to assess the activation mechanism of Torso, because the silkworm receptor expressed in the fruit-fly cells was previously confirmed to be responsive to the silkworm ligand^2^.

**Figure 1:**
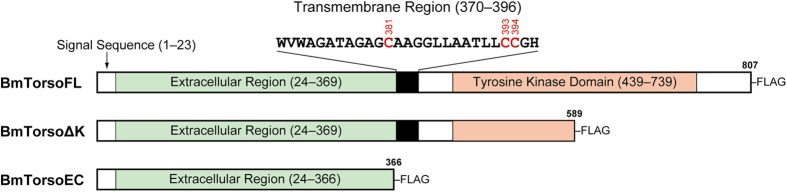
Three constructs of silkworm Torso, designed for heterologous expression in cultured *Drosophila* S2 cells. The pIZT vector was used for the expression of full-length silkworm Torso (BmTorsoFL) and the mutant that lacks the latter half of the kinase domain and its following tail (BmTorsoΔK), while the pMT vector was used for the expression of its extracellular region (BmTorsoEC). All of these proteins possessed a common secretory signal sequence at the N-terminus and an additional FLAG-tag sequence (DYKDDDDK) at the C-terminus, to facilitate detection by immunoblotting with an anti-FLAG antibody. The transmembrane regions of BmTorsoFL and BmTorsoΔK have three atypical cysteine residues, at positions 381, 393, and 394.

**Figure 2:**
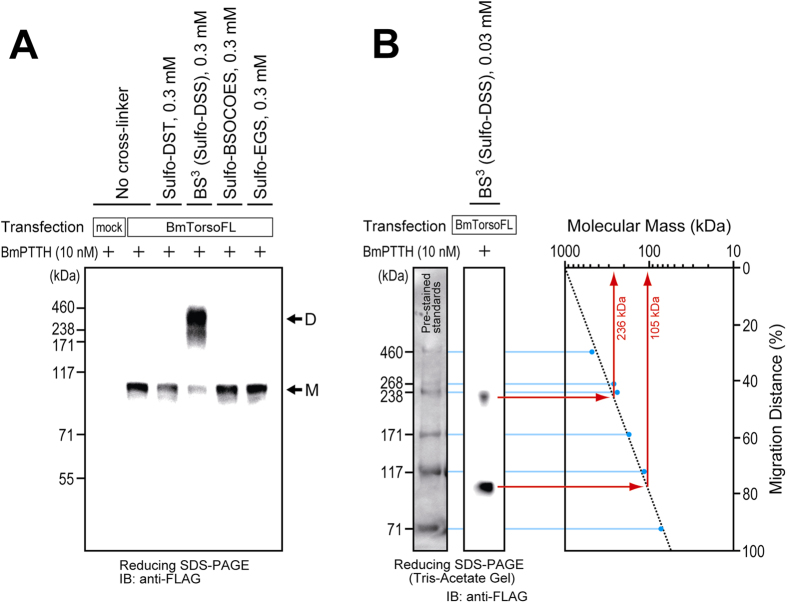
Oligomerization of silkworm Torso on the surface of cultured S2 cells. (**A**) FLAG-tagged full-length silkworm Torso (BmTorsoFL) was transiently expressed in cultured *Drosophila* S2 cells. After successive treatments with 10 nM silkworm PTTH (BmPTTH) and 0.3 mM water-soluble cross-linking reagent, the stimulated receptor was analyzed by reducing Bis-Tris SDS-PAGE. Only a cross-linker with an 11.4-Å spacer, BS^3^ (Sulfo-DSS), successfully generated an oligomer band of BmTorsoFL (shown by the arrow D), while the others, Sulfo-DST, Sulfo-BSOCOES, and Sulfo-EGS, showed only unlinked-monomer bands (the arrow M). (**B**) To estimate the precise molecular mass of the receptor complex, the BmTorsoFL-expressing cells were treated with a much lower concentration (0.03 mM) of BS^3^ and fractionated by reducing Tris-Acetate SDS-PAGE. The single logarithmic plot of the protein bands shows that the BS^3^-linked oligomer and unlinked monomer of Torso have molecular masses of 236 and 105 kDa, respectively, indicating that the BS^3^-linked complex is probably a Torso dimer.

The full-length silkworm Torso (abbreviated as BmTorsoFL) and its mutants contained a C-terminal FLAG-tag sequence when expressed in the cultured cells (Fig. 1), to facilitate detection by immunoblotting with an anti-FLAG antibody. According to the standard procedure, water-soluble sulfonated cross-linking reagents with different spacer lengths, with chemical structures shown in Fig. S2 of the Supplementary Information, were used for the detection of receptor oligomerization occurring on the surface of the cells. After the stimulation with 10 nM silkworm PTTH (BmPTTH), the cells expressing BmTorsoFL were treated with the reagents. As shown in Fig. 2A, the treatment with BS^3^ (also known as Sulfo-DSS) produced a band of the Torso oligomer on a reducing SDS-PAGE gel, while treatments with the other cross-linkers, Sulfo-DST, Sulfo-BSOCOES, and Sulfo-EGS, did not. The spacer length of BS^3^, 11.4 Å, may be suitable for trapping the Torso oligomer, represented by a smeared band centered around 240–250 kDa on the neutral-pH Bis-Tris gel. Unlinked-monomer bands were also observed at around 110 kDa, although the molecular mass of silkworm Torso is estimated as ~90 kDa, based on the amino-acid sequence. This difference in the molecular mass of the Torso monomer may result not only from the inaccuracy of the mass values estimated by the Bis-Tris SDS-PAGE, but also from the glycosylation of the receptor protein, since silkworm Torso has eight potential N-linked-glycosylation sites in its extracellular region. In addition, the oligomer band appeared at a mass 2–3 times larger than those of the monomer bands on the gel. Therefore, a Torso dimer or trimer may be trapped by the cross-linker.

To clarify the number of Torso molecules that participate in the oligomer complex, the BS^3^-linked Torso was fractionated by Tris-Acetate SDS-PAGE, which provides excellent resolution of higher molecular-mass proteins. In fact, as shown in Fig. 2B, the pre-stained standard proteins with molecular masses ranging from 71–460 kDa showed much better separation than that on the Bis-Tris SDS-PAGE gel. In addition, since an excess amount of BS^3^ provides better cross-linking efficiency but makes the linked-oligomer band smear, a much lower concentration (0.03 mM) of BS^3^ was used for the determination of the exact molecular-mass value of the Torso complex. Consequently, the molecular masses of the BS^3^-linked and unlinked receptors were estimated more precisely as 236 and 105 kDa, respectively (Fig. 2B). Thus, the BS^3^-linked Torso oligomer is probably a dimer.

For further confirmation of the Torso dimer, the formation of a hetero-oligomer complex between the full-length Torso and its truncated mutant was observed, using Tris-Acetate SDS-PAGE. The mutant, abbreviated as BmTorsoΔK, lacks the C-terminal half of the intracellular kinase domain and its following tail (Fig. 1). Although this mutant is missing half of the intracellular region of Torso, the treatment with BS^3^ revealed its oligomer band at 171 kDa upon reducing Tris-Acetate SDS-PAGE (Fig. S4A in the Supplementary Information), indicating that BmTorsoΔK maintained the oligomer formation ability. When the S2 cells were co-transfected with both vectors for the expression of BmTorsoFL and BmTorsoΔK, the unlinked-monomer and BS^3^-linked-oligomer bands were observed for both BmTorsoFL and BmTorsoΔK on the reducing gel (Fig. S4A). However, in this case, an additional band appeared at 207 kDa. This mass value is approximately the average between those of the homomeric-oligomer bands of BmTorsoFL and BmTorsoΔK, 237 and 171 kDa, respectively. This new band may be derived from the heteromeric oligomer formed between BmTorsoFL and BmTorsoΔK, since no bands other than those representing the monomers of these proteins appeared without the BS^3^ treatment. The appearance of this additional band at 207 kDa also supported the results in Fig. 2B, which showed that the oligomeric Torso complex is a dimer.

As judged from both the exact molecular mass of the Torso homomeric oligomer in Fig. 2B and that of the hetero-oligomer with the truncated mutant in Fig. S4A, the BS^3^-trapped Torso oligomer can be assigned as a *dimer*. Considering the fact that the ligand PTTH is a disulfide-bond-linked homodimer^11^, the receptor Torso could adopt a dimeric form when it binds the homodimeric ligand.

### Ligand-independent dimerization of silkworm Torso

**Figure 3:**
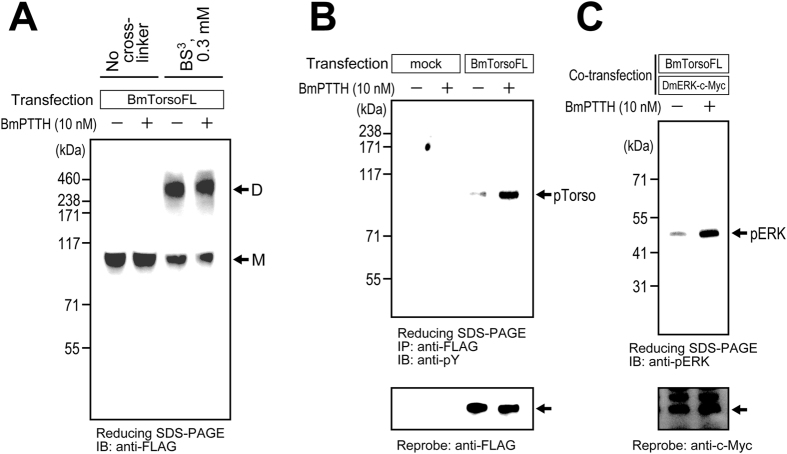
Ligand-dependency of Torso dimerization, autophosphorylation, and downstream ERK phosphorylation. (**A**) Dimerization of the FLAG-tagged full-length silkworm Torso (BmTorsoFL) was examined using the cross-linking reagent, BS^3^. The Torso dimerization was observed by immunoblotting with an anti-FLAG antibody, either with or without stimulation by the silkworm ligand PTTH (BmPTTH). (**B**) Autophosphorylation of BmTorsoFL in the membrane fractions prepared from the BmTorsoFL-expressing S2 cells was examined by immunoprecipitation with an anti-FLAG antibody and immunoblotting with an anti-phosphotyrosine antibody. The Torso autophosphorylation (shown by the arrow pTorso) was promoted by the BmPTTH stimulation. (**C**) Phosphorylation of the ERK enzyme downstream of Torso was examined in the BmTorsoFL-expressing S2 cells, by immunoblotting with an anti-phospho-ERK antibody. ERK phosphorylation (the arrow pERK) was robustly increased by the BmPTTH stimulation. Since an antibody specifically recognizing the fruit-fly ERK (DmERK) was not available, c-Myc-tagged ERK (DmERK-c-Myc) was co-expressed in the cells, as a protein loading control.

Next, the ligand-dependency of Torso dimerization was assessed using the cross-linker, BS^3^. Surprisingly, as shown in Fig. 3A, the Torso dimer band was also detected without the PTTH stimulation. Torso may naturally exist as a dimer on the cellular membrane, before the ligand stimulation.

Since the receptor dimerization was revealed to occur independently of the ligand stimulation, the ligand-dependency of the Torso autophosphorylation was then assessed. Membrane fractions were prepared from serum-starved S2 cells, which transiently expressed the BmTorsoFL protein. After an incubation with or without 10 nM BmPTTH for 10 min, the receptor in the membrane fractions was solubilized with 1% Triton-X and 0.5% sodium deoxycholate, and then immunoprecipitated with an anti-FLAG antibody. Torso was separated by reducing SDS-PAGE, and autophosphorylation was detected by immunoblotting with an anti-phosphotyrosine antibody (Fig. 3B). When stimulated by the recombinant PTTH, much higher levels of Torso autophosphorylation were observed, as compared to that without the stimulation. In addition, in the BmTorsoFL-expressing S2 cells, intracellular ERK phosphorylation was robustly increased in response to the ligand stimulation (Fig. 3C), as reported previously^2^. These results indicate that the activation of the receptor Torso is dependent on the stimulation by the ligand PTTH, even though Torso adopts the dimeric form before the stimulation.

### Disulfide-bond-mediated dimerization of silkworm Torso

**Figure 4:**
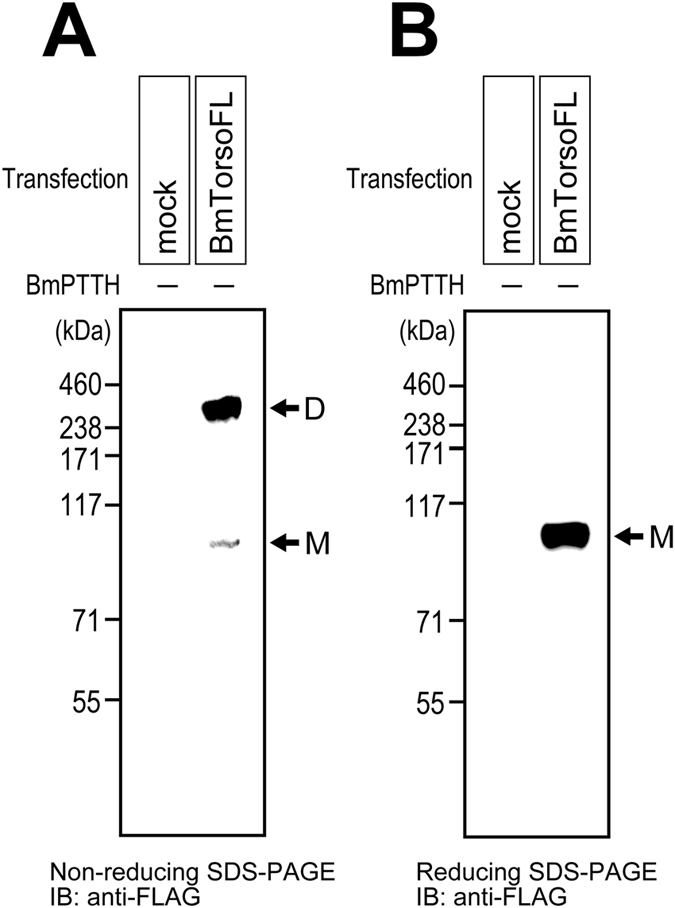
Disulfide-bond-mediated dimerization of silkworm Torso without ligand stimulation. FLAG-tagged full-length silkworm Torso (BmTorsoFL), expressed in cultured S2 cells, was analyzed by non-reducing (**A**) and reducing (**B**) Bis-Tris SDS-PAGE, without the cross-linking treatment. Even without the BmPTTH stimulation, Torso was detected as an apparent dimer band (shown by the arrow D), in addition to a faint monomer band (the arrow M), on the non-reducing gel, while it was only detected as the monomer band on the reducing gel.

To reveal the association mode of the receptor dimer, BmTorsoFL was examined by non-reducing SDS-PAGE. If the Torso molecules in the dimer are linked by intermolecular disulfide bridges, then the dimer band might be observed on a non-reducing gel, without the cross-linking treatment. After the BmTorsoFL-expressing S2 cells were solubilized directly in SDS-PAGE sample buffer without the reductant, the proteins were fractionated by non-reducing Bis-Tris SDS-PAGE (Fig. 4A). Visualization by immunoblotting with an anti-FLAG antibody revealed a clear dimer band at ~240 kDa, in addition to a faint monomer band at ~100 kDa. Since the sample prepared by the same procedure showed a single band at ~110 kDa upon reducing Bis-Tris SDS-PAGE (Fig. 4B), the ~240-kDa band on the non-reducing gel may be derived from the disulfide-bond-linked Torso dimer, although it seemed to be slightly smaller than the BS^3^-linked dimer (240–250 kDa) on the reducing gel (Fig. 2A). Since the BS^3^-linked Torso dimer also appeared at around 240 kDa on the non-reducing gel (data not shown), this slight mass difference between the dimers may arise from the redox conditions in the Bis-Tris SDS-PAGE. In fact, on the non-reducing gel, the faint monomer band was observed at a smaller mass (~100 kDa) than that observed on the reducing gel (~110 kDa) (Fig. 4). Since the dimer band was completely undetectable on the reducing gel, the Torso dimer is constructed by one or more intermolecular disulfide bridge(s).

### The extracellular cysteines do not participate in the intermolecular disulfide bridges

**Figure 5:**
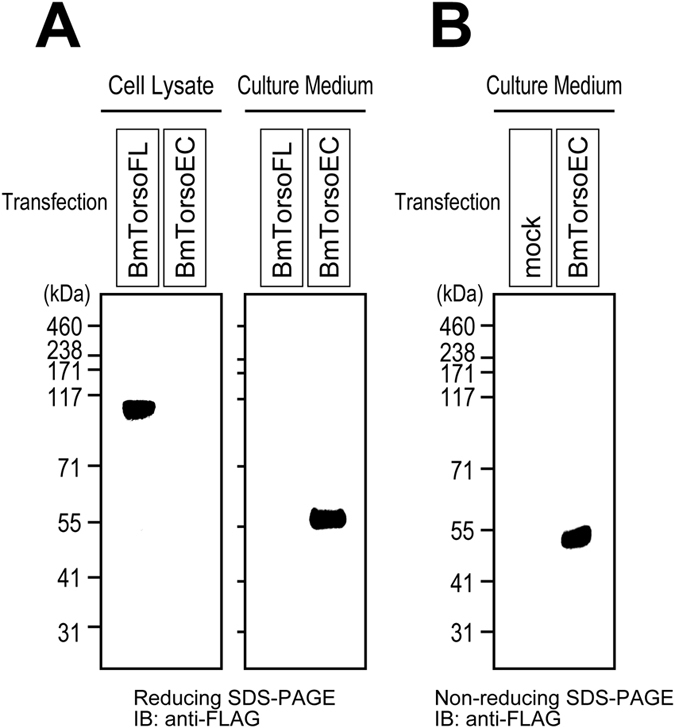
Expression and oligomerization states of the silkworm Torso extracellular region. (**A**) FLAG-tagged full-length silkworm Torso (BmTorsoFL) and its extracellular region (BmTorsoEC), expressed in cultured S2 cells, were analyzed by reducing Bis-Tris SDS-PAGE. While BmTorsoFL was found in the cell lysate, BmTorsoEC was not detected in the lysate, but was present in the culture medium of the cells. (**B**) The BmTorsoEC secreted in the culture medium was not detected as the dimer band on the non-reducing Bis-Tris gel.

The mature form of silkworm Torso has eight cysteine residues in its extracellular region (Fig. S1), and all of these cysteines may potentially participate in the intra- or inter-molecular disulfide bridges under the oxidative culture conditions in Schneider’s medium. To identify the cysteine residues that participate in the intermolecular bridges, we prepared another truncated Torso mutant, composed of only the extracellular region (BmTorsoEC in Fig. 1). The mutant was transiently expressed in cultured S2 cells, and then both the whole cell lysate and culture medium were subjected to reducing Bis-Tris SDS-PAGE. Immunoblotting with an anti-FLAG antibody revealed the presence of BmTorsoEC in the medium, but not in the cell lysate, while BmTorsoFL was detected in the lysate, but not in the medium (Fig. 5A). The extracellular mutant was secreted into the culture medium because it lacked the anchoring transmembrane region, when it was expressed with the N-terminal secretory signal sequence. Since the molecular mass of BmTorsoEC is estimated as ~40 kDa, based on its amino-acid sequence, the secreted BmTorsoEC, with a band appearing at ~58 kDa on reducing SDS-PAGE, may also be glycosylated. Since no bands were observed at higher molecular masses than the monomer band at ~52 kDa on the non-reducing gel (Fig. 5B), the Torso extracellular mutant did not adopt the dimeric form. Therefore, none of the eight extracellular cysteines participate in the intermolecular bridges connecting the two receptor molecules in the Torso dimer, although they form intramolecular disulfide bridges within the receptor polypeptide chain.

### Intermolecular disulfide bridges are formed between the cysteine residues in the transmembrane region

In contrast to the extracellular mutant of silkworm Torso (BmTorsoEC), the mutant lacking half of the intracellular region (BmTorsoΔK) appeared as a dimer band on a non-reducing gel, without the cross-linking treatment (Fig. S4B in the Supporting Information). Therefore, the cysteine residues participating in the intermolecular disulfide bridges may exist in the region that was present in BmTorsoΔK, but not in BmTorsoEC. As shown in Fig. 1, the transmembrane region of silkworm Torso has three cysteine residues at positions 381, 393, and 394, although cysteines are rarely found in this region in other single-pass transmembrane proteins. These cysteines may participate in the intermolecular bridges. In fact, the non-sulfonated cross-linkers, DSS and BSOCOES, trapped the Torso dimer more efficiently than their corresponding sulfonated versions, BS^3^ and Sulfo-BSOCOES, respectively (Fig. S3). Since the non-sulfonated reagents are more accessible to the hydrophobic cellular-membrane surface than the sulfonated ones^17^,^18^,^19^, the amino groups that are intermolecularly cross-linked by the reagents may be located in the juxtamembrane regions, which could be brought closer to each other by the disulfide bridges in the transmembrane region.

**Figure 6:**
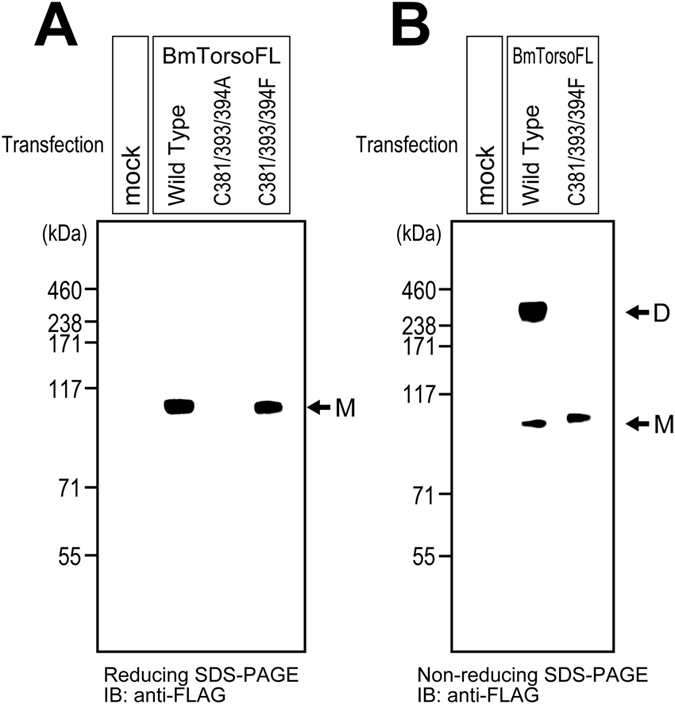
Expression and oligomerization states of silkworm Torso mutants, in which three cysteine residues in the transmembrane region were replaced by alanines (C381/393/394A) and phenylalanines (C381/393/394F). After transfection with a vector for the expression of wild-type Torso or its substitution mutant, the S2 cells without the ligand stimulation were solubilized and directly analyzed by reducing (**A**) and non-reducing (**B**) Bis-Tris SDS-PAGE. The wild-type and phenylalanine mutant proteins were observed as similar monomer bands (shown by the arrow M) on the reducing gel, while that of the alanine mutant was not detected. Wild-type Torso was detected as both the monomer and dimer bands (the arrows M and D, respectively) on the non-reducing gel, while the phenylalanine mutant was only detected as the monomer.

To clarify the contributions of the three cysteines in the transmembrane region to the disulfide-bond-linked dimerization, we designed additional Torso mutants, in which the three residues were replaced by alanines (C381/393/394A) or phenylalanines (C381/393/394F). After the transfection with an expression vector for each mutant, the cultured S2 cells were solubilized directly in SDS-PAGE sample buffer. The Torso proteins were fractionated by reducing SDS-PAGE, and were visualized by immunoblotting with an anti-FLAG antibody. As shown in Fig. 6A, when the three transmembrane cysteine residues were replaced by alanines, the mutant (C381/393/394A) was not detected in the cell lysate, while the parent wild-type Torso was detected in the lysate and appeared at ~110 kDa on the reducing gel. Since the alanine mutant was not detected in the culture medium (data not shown), the expressed mutant receptor may be degraded in the cells. The substitution with residues that are more hydrophilic than the original cysteines in the transmembrane region might have destabilized the receptor molecule.

Therefore, the three cysteines were replaced by more hydrophobic amino acids, phenylalanines. The expression of the phenylalanine mutant (C381/393/394F) was confirmed on a reducing Bis-Tris SDS-PAGE gel, as a similar monomer band to that of the wild-type receptor, at ~110 kDa (Fig. 6A). However, on a non-reducing gel, while the wild-type BmTorsoFL showed both a stronger dimer band at ~240 kDa and a faint monomer band at ~100 kDa, the phenylalanine mutant showed only a monomer band at 100–110 kDa (Fig. 6B). Since no dimer band of the phenylalanine mutant appeared, the cysteine residues in the transmembrane region are responsible for the intermolecular disulfide bridges in the Torso dimer.

### Autophosphorylation, dimerization, and downstream activation by the Torso phenylalanine mutant

To elucidate the role of the intermolecular disulfide bridges in the receptor functions, the autophosphorylation, dimerization, and downstream activation by the phenylalanine mutant (C381/393/394F) were examined.

**Figure 7:**
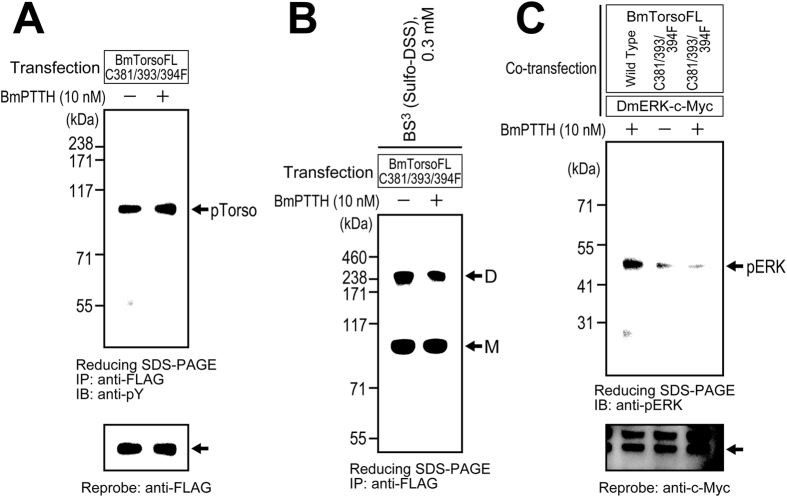
Receptor autophosphorylation, dimerization, and downstream ERK phosphorylation of the Torso phenylalanine mutant (C381/393/394F). The receptor autophosphorylation (**A**), dimerization (**B**), and downstream ERK phosphorylation (**C**) by the phenylalanine mutant were assessed in the same manner as described in Fig. 3. The mutant receptor was similarly autophosphorylated either with or without the ligand stimulation, and it formed a non-covalent dimer independently of the ligand stimulation. However, ERK phosphorylation was not facilitated by ligand stimulation in the phenylalanine-mutant-expressing S2 cells, while it was promoted by the ligand in those expressing wild-type Torso (positive control).

First, the ligand-dependent autophosphorylation of the mutant receptor was assessed. After transfection with the expression vector encoding the phenylalanine mutant, the S2 cells were treated in the same manner as in Fig. 3B. Surprisingly, the mutant was highly autophosphorylated both before and after the ligand stimulation (Fig. 7A), although its disulfide-bond-linked dimerization was not observed (Fig. 6B). Since the autophosphorylation of single-polypeptide RTKs, such as the members of subclass-I and III RTKs, is always triggered by their oligomerization, the dimerization of the phenylalanine mutant was re-examined using the cross-linking reagent, in the same manner as in Fig. 3A. As shown in Fig. 7B, BS^3^-linked-dimer bands were obviously detected at ~240 kDa, both with and without the PTTH stimulation. These data indicated that the Torso phenylalanine mutant spontaneously forms a non-covalently-associated dimer on the cellular membrane, independently of ligand stimulation, and the dimerization causes the ligand-independent autophosphorylation of the receptor. The disulfide bridges in the transmembrane region may function to prevent the receptor from unregulated dimerization and autophosphorylation occurring without PTTH stimulation.

Next, the intracellular ERK phosphorylation through the autophosphorylation of the phenylalanine mutant (C381/393/394F) was assessed, in the same manner as in Fig. 3C. As shown in Fig. 7C, ERK was hardly phosphorylated even after the PTTH stimulation, although it was robustly phosphorylated in the wild-type Torso-expressing cells (positive control). These data indicated that the unregulated autophosphorylation in the phenylalanine mutant cannot induce the ERK phosphorylation in the downstream pathway of the cells. By preserving the proper relative positioning of the two receptor molecules, the intermolecular disulfide bridges in the transmembrane region may minimize the unregulated autophosphorylation of Torso and maintain the ligand-dependent regulation of the cellular activities.

## Discussion

The extracellular region of Torso adopts a unique primary structure, which shares quite low similarity with those of other RTK receptors. In addition, silkworm Torso has three atypical cysteine residues in its transmembrane region. In this study, these cysteines were revealed to participate in intermolecular disulfide bridges, which maintain the ligand-responsive properties of the receptor molecules, although it remains to be clarified which cysteines among the three residues actually form the intermolecular disulfide bridges. Insulin receptor also forms an α_2_β_2_-heterotetramer complex combined through intermolecular disulfide bridges, but all of the bridges exist in the extracellular regions. In contrast, the intermolecular disulfide bridges of silkworm Torso were found in the transmembrane region. Such disulfide-bond-mediated linkage in the transmembrane region makes it difficult to classify Torso into any of the three major RTK subclasses.

**Figure 8:**
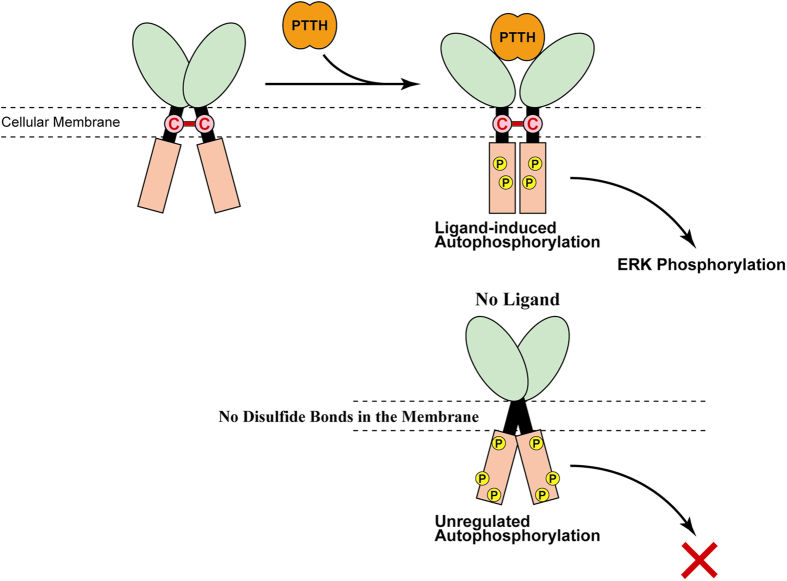
Possible model for the role of the disulfide bridges in the transmembrane region of Torso. In wild-type Torso (upper case), PTTH binding to the extracellular region triggers phosphorylation in the intracellular region by its own kinase activity, and the autophosphorylation facilitates ERK phosphorylation in the downstream signaling pathway. In contrast, without the disulfide bridges in the transmembrane region, as in the phenylalanine mutant (C381/393/394F) (lower case), Torso spontaneously forms a non-covalently-associated dimer, rather than a disulfide-bond-mediated dimer, on the cellular membrane even in the absence of the ligand, and the dimerization causes the ligand-independent receptor autophosphorylation. Although the phosphorylation sites were not identified in this study, the sites in the unregulated, ligand-independent autophosphorylation of the receptor lacking the intermolecular linkage may be different from those in the ligand-dependent phosphorylation of the wild-type Torso, because the unregulated autophosphorylation cannot facilitate downstream ERK phosphorylation. The intermolecular disulfide bridges in the transmembrane region maintain the normal ligand-dependent receptor functions, by preserving the proper relative positioning of the two Torso molecules.

Cysteines readily form disulfide bridges under the oxidative extracellular conditions, while they remain unoxidized and retain free sulfhydryl groups under the reductive intracellular conditions. Presently, little is known about the redox conditions in the cellular membrane. There are several membrane protein complexes in which the protein molecules are connected by disulfide bridges in their transmembrane region. For example, neurotrophin receptor, p75^NTR^, is a dimeric protein in which the two receptor molecules are linked by a disulfide bridge in the transmembrane region^20^. This receptor is not an RTK-family member, but like Torso, it is composed of a single-membrane-spanning polypeptide, and adopts a homodimeric form independently of ligand stimulation. In the case of p75^NTR^, the intermolecular disulfide bridge in the transmembrane region is thought to function as a hinge to transduce the ligand-driven conformational changes in the extracellular region into the movement of the intracellular domains that recruit several adaptor proteins. In contrast, in the case of Torso, the disulfide bridges in the transmembrane region may reduce the unregulated dimerization and subsequent autophosphorylation, by maintaining the proper positioning of the two receptor molecules (Fig. 8). Similar suppressive regulation to restrain unregulated receptor activation has also been found in other RTK receptors. For example, in the case of human EGFR, spontaneous dimerization without ligand stimulation is hampered by the collision of the large globular extracellular domains of the subunits, because the deletion of the receptor ectodomains resulted in the receptor autophosphorylation^9^ and the rapid proliferation of the receptor-expressing cultured cells^21^. Since the extracellular region of silkworm Torso is much smaller (~350 amino-acid residues) than that of EGFR (~620 residues), the disulfide bridges in the transmembrane region may alternatively prevent the Torso molecules from non-covalent dimerization occurring without ligand stimulation.

Even though the removal of the intermolecular disulfide bridges from the transmembrane region caused the spontaneous, ligand-independent autophosphorylation of Torso, this unregulated autophosphorylation did not facilitate ERK phosphorylation in the downstream signaling pathway. This difference in the activation ability of the downstream pathway may be derived from a structural difference between the disulfide-bond-mediated dimer and the non-covalently-associated dimer (Fig. 8). In fact, a structural difference between the dimers was observed in the cross-linking experiment. As shown in Fig. S5, the non-covalently-associated dimer of the phenylalanine mutant was trapped with Sulfo-EGS, while the disulfide-bond-mediated dimer of the wild-type Torso was not. This structural difference may cause a difference in the intracellular autophosphorylation sites between the dimers.

In fruit-fly Torso, the autophosphorylation sites were previously assigned to six tyrosine residues in the intracellular region^22^. Among the six residues, two tyrosines, Y767 and Y772, are involved in the so-called activation loop of the tyrosine kinase domain, and the phosphorylation of the tyrosines in the loop often triggers the promotion of its own kinase activity. Actually, mutations of the tyrosine residues to phenylalanines profoundly reduced the autophosphorylation of fruit-fly Torso^22^. The tyrosine residues Y633 and Y638 in silkworm Torso, which correspond to Y767 and Y772 in the fruit-fly receptor, respectively, may also be phosphorylated in both the ligand-induced and unregulated autophosphorylation. In contrast, Y541 or Y542 in silkworm Torso, corresponding to Y630 in fruit-fly Torso, may not be phosphorylated in the unregulated autophosphorylation. However, the residues may become phosphorylated in the ligand-induced autophosphorylation, because Y630 of fruit-fly Torso is reportedly a recruiting site for an adaptor protein, Corkscrew, which is located upstream of the ERK enzyme in the intracellular signaling pathway^23^,^24^. Since several other tyrosines in fruit-fly Torso became phosphorylated by ligand stimulation^22^, the unregulated autophosphorylation in the phenylalanine mutant could potentially activate intracellular enzymes other than ERK, without ligand stimulation. Therefore, such ligand-independent autophosphorylation should be avoided, to maintain the normal regulation of insect development induced by PTTH. Identification of the autophosphorylation sites in silkworm Torso is one of the most important subjects to address, and the differences in the phosphorylation sites between the wild-type Torso and the phenylalanine mutant may explain the differences in their abilities to activate the downstream intracellular pathways.

The ligand PTTH exhibits quite high target specificity between insect species. The hormones from *Bombyx mori* and *Manduca sexta* induced adult development of the same insect species, but not of the different one^25^,^26^. Torso must recognize the differences in the PTTH structures between the insect species. The dimerization through the disulfide bridges in the transmembrane region may also contribute to the precise ligand recognition by Torso, by constructing a wider binding site for the quite large ligand PTTH (homodimer composed of two ~110-residue polypeptides).

In conclusion, although Torso is a single-polypeptide RTK, like the members of subclass-I and III RTKs, it forms a dimer linked by intermolecular disulfide bridges, as found in the members of subclass-II RTKs, such as the insulin receptor. Torso may transduce the PTTH stimulation into the intracellular autophosphorylation through conformational changes, as in the insulin receptor, rather than through further oligomerization. However, while all the intermolecular disulfide bridges of the insulin receptor exist in the extracellular region, those of the silkworm Torso connect the two receptor molecules by linking the atypical cysteine residues in the transmembrane region. This unique structure is very important for Torso to maintain the ligand-dependent properties of its receptor functions.

## Methods

### Cloning of the silkworm Torso gene

The silkworm (*Bombyx mori*, p50T laboratory strain) larvae were graciously provided by Dr. Yutaka Banno (Institute of Genetic Resources, Faculty of Agriculture, Kyushu University). Total RNA was isolated from the prothoracic glands of the larvae in the wandering stage, using an RNeasy Mini Kit (QIAGEN, Venlo, The Netherlands). After on-column digestion of the contaminating DNA with DNase I (QIAGEN), the purified RNA was reverse-transcribed into cDNA with an Oligo(dT)_18_ Primer (Fermentas, Vilnius, Lithuania) and SuperScript^®^ III Reverse Transcriptase (Invitrogen Corporation, Carlsbad, CA, USA). The silkworm Torso cDNA was amplified from the prepared total cDNA by standard polymerase chain reactions (PCRs), using TaKaRa Ex Taq^®^ (Takara Bio, Ohtsu, Shiga, Japan). The primers used in this study are listed in Table S1 of the Supplementary Information. The amplified cDNA fragments were purified using a Wizard^®^ SV Gel and PCR Clean-Up System (Promega Corporation, Madison, WI, USA), and then introduced into the pGEM^®^-T Easy Vector (Promega Corporation). After amplification in *E. coli* DH5α cells, the vector was extracted using a FastGene Plasmid Mini Kit (Nippon Genetics Co, Ltd., Tokyo, Japan). The nucleotide sequence of the silkworm Torso gene was analyzed on a 3130xl Genetic Analyzer by a PCR protocol using a BigDye^®^ Terminator v3.1 Cycle Sequencing Kit (Applied Biosystems, Inc., Carlsbad, CA, USA). The sequences of the silkworm Torso genes were completely identical among the four cloned vectors, but nine amino-acid residues encoded by the genes were different from those published in the UniProt Knowledge Base (Entry name: D2IYS2_BOMMO): L8S, K9R, V73I, K177Q, H431R, E487K, S528C, D547N, and S600A. These variations in the amino-acid sequence may be derived from the differences in the silkworm strains.

### Construction of expression vectors for silkworm Torso and its mutants

To prepare expression vectors for BmTorsoFL, BmTorsoΔK, and the cysteine-substitution mutants of BmTorsoFL (C381/393/394A and C381/393/394F), the genes were manipulated and amplified by PCR, using KOD -plus- or KOD FX DNA polymerase (TOYOBO Co., Ltd., Osaka, Japan). The DNA fragments, possessing an additional sequence encoding the C-terminal FLAG-tag, were introduced into a modified pIZT/V5-His vector (Invitrogen Corporation). The BmTorsoEC gene was similarly introduced into the pMT/V5-His A vector (Invitrogen Corporation), which was developed for higher heterologous expression in cultured *Drosophila* S2 cells. Since BmTorsoEC was produced as a secreted protein and diluted in the culture medium, a more efficient expression system was required for its detection to be comparable to that of the other Torso proteins.

### Expression of the Torso proteins

For the transient expression of the Torso proteins, we used cultured *Drosophila* S2 cells, which were a component of the DES^®^ Inducible Kit (Life Technologies, Inc., Carlsbad, CA, USA). The cells were transfected with the expression vector or the corresponding empty vector (mock), using the Lipofectamine^®^ LTX Reagent (Life Technologies, Inc.). After the transfection, the S2 cells were cultured at 25 °C for 24 h in Schneider’s *Drosophila* medium, supplemented with fetal bovine serum (FBS; AusGeneX Pty Ltd., Molendinar, QLD, Australia) and antibiotics. In the case of the Torso proteins other than BmTorsoEC, the cells were starved in serum-free medium for an additional 24 h, to reduce the background activities possibly stimulated by serum components. For the production of BmTorsoEC, the cells were cultured in serum-free medium containing 0.5 mM CuSO_4_ for an additional 48 h to induce protein production, because the metal-ion-sensitive *Drosophila* metallothionein promoter is employed in the pMT vector system.

### Ligand PTTH preparation

The recombinant silkworm PTTH (BmPTTH) was prepared by the *Brevibacillus* secretory expression system^12^,^13^, with a C-terminal His_6_-tag sequence. A brief outline of the preparation method was previously published^27^, and detailed expression and purification conditions will be reported separately.

### SDS-PAGE and immunoblotting

Unless otherwise described, all expressed proteins were analyzed by neutral-pH Bis-Tris SDS-PAGE (NuPAGE^®^ Bis-Tris gels, Life Technologies, Inc.) to avoid undesired disulfide-bond rearrangement during the electrophoresis^28^, although the molecular-mass values estimated from the Bis-Tris gels are usually less accurate than those from the standard alkaline Laemmli Tris-Glycine gels^29^. The Torso proteins were fractionated using 4–12% Bis-Tris gels with a MOPS buffer system, while the extracellular mutant BmTorsoEC shown in Fig. 5 was resolved using 12% Bis-Tris gels with a MES buffer system. For more precise estimations of the molecular masses of the Torso oligomer complexes under reducing SDS-PAGE conditions, NuPAGE^®^ Novex^®^ 3–8% Tris-Acetate Protein Gels (Life Technologies, Inc.) were employed, because the wider measurement range of the gels allows better separation of larger proteins^30^.

The separated proteins on the SDS-PAGE gel were transferred onto a FluoroTrans^®^ PVDF membrane (Pall Corporation, Pensacola, FL, USA) for immunoblotting. The primary antibodies were a monoclonal anti-FLAG antibody (mouse, #M185-3S, Lot. 002, Medical & Biological Laboratories Co., Ltd., Nagoya, Japan), a monoclonal anti-phosphotyrosine antibody (PY99) (mouse, #sc-7020, Lot. G0913, Santa Cruz Biotechnology, Inc., Santa Cruz, CA, USA), a polyclonal anti-[phospho-p44/42 MAPK (erk1/2) (Thr202/Tyr204)] antibody (rabbit, #9101, Lot. 27, Cell Signaling Technology, Inc., Danvers, MA, USA), and a monoclonal anti-c-Myc antibody (9E10) (mouse, #sc-40, Lot. B1313, Santa Cruz Biotechnology, Inc.). Protein bands were visualized by ImmunoStar^®^ LD (Wako Pure Chemical Industries, Ltd., Osaka, Japan) with horse-radish peroxidase (HRP)-conjugated secondary anti-mouse IgG and anti-rabbit IgG antibodies (#7076 and #7074, respectively, Cell Signaling Technology, Inc.). All immunoblotting data in this paper were acquired at least three times, and typical data are presented in the figures.

### Cross-linking experiments

Cross-linking reagents, Sulfo-DST, BS^3^, Sulfo-EGS, and DSS, were purchased from Thermo Fisher Scientific, Inc. (Waltham, MA, USA). Sulfo-BSOCOES was obtained from INDOFINE Chemical Company, Inc. (Hillsborough, NJ, USA), and BSOCOES was from Fluka Chemie AG (Buches, Switzerland). BS^2^G was purchased from ProteoChem (Loves Park, IL, USA).

The S2 cells expressing the Torso proteins were collected by centrifugation at 700 × *g*, at 4 °C for 15 min, washed three times with 20 mM HEPES buffer (pH 7.4), and re-suspended in the same buffer. After an incubation with or without 10 nM BmPTTH for 10 min, the cells were treated with a cross-linking reagent for 10 min. The concentration of the cross-linking reagents was usually 0.3 mM, but it was reduced to 0.03 mM for the precise estimation of the molecular masses of the cross-linked complexes. After termination of the cross-linking reaction with 50 mM glycine, the cells were solubilized directly in SDS sample buffer containing 0.5 M DTT, for analyses by reducing SDS-PAGE.

### Autophosphorylation assay

The S2 cells expressing the Torso proteins were sonicated, and the membrane fractions were prepared from the cell homogenate by ultracentrifugation at 103,000 × *g*, at 4 °C for 90 min, and dispersed in resuspension buffer (1:1 v/v solution of HEPES-buffered saline (HBS) and Schneider’s *Drosophila* medium). After an incubation with or without 10 nM BmPTTH for 10 min, the fractions were solubilized on ice for 15 min in resuspension buffer, containing 1% Triton X-100, 0.5% sodium deoxycholate, 5 mM EDTA, 1 mM PMSF, and a Phosphatase Inhibitor Cocktail (Nacalai Tesque, Inc., Kyoto, Japan). The cell debris was removed by centrifugation at 20,000 × *g*, at 4 °C for 5 min, and the supernatant was subjected to immunoprecipitation with a monoclonal anti-FLAG antibody (mouse, #M185-3S, Lot. 002, Medical & Biological Laboratories Co., Ltd.) at 4 °C for 1 h, and with Protein A/G PLUS-Agarose (#sc-2003, Santa Cruz Biotechnology, Inc.) for an additional hour. The immunoprecipitates were washed three times with HBS containing 1% Triton X-100 and 0.5% sodium deoxycholate. The precipitated proteins were eluted with 150 μg/ml DYKDDDDK Peptide (Wako Pure Chemical Industries, Ltd.) and subjected to reducing SDS-PAGE. The autophosphorylation was determined by immunoblotting with an antibody that specifically recognizes phosphorylated tyrosine residues.

### ERK phosphorylation assay

Since an antibody against *Drosophila* ERK (DmERK) was not commercially available, the DmERK possessing a C-terminal c-Myc-tag sequence was co-expressed with the Torso proteins, as a loading control. The DmERK gene was obtained from the S2 cells by a similar method to that used for obtaining silkworm *torso*, as described above, and it was manipulated and introduced into the modified pIZT/V5-His vector.

After the S2 cells were co-transfected with the expression vectors for the silkworm Torso (BmTorsoFL or its phenylalanine mutant) and DmERK-c-Myc, they were collected and resuspended in the same resuspension buffer described above. After an incubation with or without 10 nM BmPTTH for 20 min, the cells were solubilized on ice for 15 min with HBS, containing 1% Triton X-100, 0.5% sodium deoxycholate, 5 mM EDTA, 1 mM PMSF, and a Phosphatase Inhibitor Cocktail (Nacalai Tesque, Inc.). The cell debris was removed by centrifugation at 20,000 × *g*, at 4 °C for 5 min, and the supernatant was diluted with the same buffer to 1 μg/μl total protein concentration. The proteins were resolved by reducing SDS-PAGE, using 4–12% Bis-Tris SDS-PAGE gels in combination with a MES buffer system. The ERK activation was determined by immunoblotting with an antibody that specifically recognizes phosphorylated ERK, and the loading control was estimated by reprobing the DmERK-c-Myc blot with an anti-c-Myc antibody.

## Additional Information

**How to cite this article**: Konogami, T. *et al.* Ligand-dependent responses of the silkworm prothoracicotropic hormone receptor, Torso, are maintained by unusual intermolecular disulfide bridges in the transmembrane region. *Sci. Rep.* **6**, 22437; doi: 10.1038/srep22437 (2016).

## Supplementary Material

Supplementary Information

Supplementary Information
