# Supplementary material for: Ligand-dependent responses of the silkworm prothoracicotropic hormone receptor, Torso, are maintained by unusual intermolecular disulfide bridges in the transmembrane region
Source: Sci Rep. 2016 Mar 1;6:22437. doi: 10.1038/srep22437 (PMC4772477; doi:10.1038/srep22437)
Supplement: Supplementary Information [file srep22437-s1.pdf]

Supplementary Information

Ligand-dependent responses of the silkworm prothoracicotropic hormone receptor, Torso, are maintained by unusual intermolecular disulfide bridges in the transmembrane region

Tadafumi Konogami, Yiwen Yang, Mari H. Ogihara, Juri Hikiba, Hiroshi Kataoka & Kazuki Saito

1. Uniqueness of the Torso extracellular region

In terms of its extracellular domain composition, Torso is potentially classified into RTK subclass III, because it lacks a cysteine-rich domain, and the absence of the extracellular cysteine-rich domain is a major characteristic feature of the subclass-III RTKs. In Fig. S1, the extracellular amino-acid sequences of the silkworm (*Bombyx mori*) and fruit-fly (*Drosophila melanogaster*) Torso proteins (abbreviated as BmTorso and DmTorso, respectively) are listed along with those of typical members of the subclass-III RTKs. PDGFRα (hPDGFRA), VEGFR type-1 (hVEGFR1), and FGFR type-1 (hFGFR1) are typical subclass-III members of human RTKs. DmPVR was identified as a PDGFR/VEGFR homologue in fruit fly, while DmBTL and DmHTL were found as FGFR homologues.

In Fig. S1, only the N-terminal signal-peptide (shown by the yellow boxes in Fig. S1) and extracellular regions of the receptors are cited. The extracellular regions are estimated, based on descriptions in the UniProt Knowledge Base [UniProt entries: BmTorso, D2IYS2; DmTorso, P18475; DmPVR, Q95P10; DmBTL, Q09147; DmHTL, Q07407; hPDGFRA, P16234; hVEGFR1, P17948; hFGFR1, P11362]. However, in the cases lacking this information, the extracellular region was assumed to be an intermediate part between the signal sequence and the transmembrane region, which were predicted by the 'SignalP 4.1 Server' [S1] and the 'TMHMM Server v. 2.0' [S2], respectively. In Fig. S1, cysteine residues are colored red, and disulfide bonds that were defined in hFGFR1 [S3] are shown by red lines.

Neither silkworm nor fruit-fly Torso has a cysteine-rich domain, and their extracellular sequences are quite different from those of the subclass-III RTK members listed in Fig. S1. One of the main differences is the absence of immunoglobulin-like (Ig-like) domains (shown by blue boxes), which mostly possess conserved cysteine residues. The typical human subclass-III RTKs have several Ig-like domains in their extracellular regions, and such Ig-like domains are also present in some insect RTKs. Neither silkworm nor fruit-fly Torso has an Ig-like domain, although another fibronectin type 3 (FN3) domain is predicted to exist in the extracellular region of fruit-fly Torso (shown by a green box). Even though both BmTorso and DmTorso lack the extracellular cysteine-rich domain, their extracellular structures are too unique to classify the receptors into RTK subclass III.

2. Cross-linking reagents used to study Torso oligomerization

Since succinimidyl esters react with the free amino groups of proteins, such as the N-terminal α-amino groups and the side-chain ε-amino groups of lysine residues, disuccinimidyl esters with a spacer chain are often utilized for trapping a protein complex, by linking the groups from the different protein molecules forming the complex. However, since a suitable spacer length of the linker must be selected for bridging such amino groups at a distance, several reagents with different spacer lengths should be tested for performing the cross-linking experiments efficiently. In this study, we used four disuccinimidyl esters, with chemical structures and spacer lengths listed in Fig. S2, to study Torso oligomerization (Fig. 2A). To reveal the receptor oligomerization occurring on the extracellular surface of the cells, water-soluble sulfonated cross-linkers were primarily employed, because they cannot penetrate the hydrophobic cellular membrane[18,19].

We also used the non-sulfonated versions of BS^3 (Sulfo-DSS) and Sulfo-BSOCOES, which were the most efficient and somewhat less efficient reagents to trap the BmTorsoFL oligomer complex, respectively, in Fig. 2A. Since DSS and BSOCOES share the same structures with BS^3 and Sulfo-BSOCOES, respectively, but lack the hydrophilic sulfonate groups ($-SO_3Na$) in the succinimidyl parts at both ends, these reagents are more accessible to the hydrophobic membrane surface of the cells. As shown in Fig. S3, DSS produced stronger dimer bands (shown by the arrow D) than BS^3, either with or without the PTTH stimulation. In addition, BSOCOES generated weak dimer bands, whereas Sulfo-BSOCOES showed only the unlinked Torso monomer bands (the arrow M), but not the dimer bands. Such increased cross-linking efficiencies by the non-sulfonated reagents indicate that the linkers may bridge the side chains of the lysine residues, located in the juxtamembrane regions of the Torso molecules in the dimer. The juxtamembrane regions may become close enough to be linked, due to the intermolecular disulfide bridges in the transmembrane region.

3. Oligomerization of a silkworm Torso mutant lacking half of the intracellular region (BmTorsoΔK)

For further confirmation of the Torso dimer formation, the full-length receptor (BmTorsoFL) and the truncated mutant (BmTorsoΔK) were co-expressed in cultured S2 cells. If the BS^3-trapped Torso complex in Fig. 2A is a dimer, then a band representing a heterodimer between BmTorsoFL and BmTorsoΔK (FL–ΔK) will appear at an average molecular mass between those of their homodimers, FL_2 and ΔK_2. In contrast, if the complex is a trimer, then two kinds of heterotrimers, FL_2–ΔK and FL–ΔK_2, will be newly observed between the bands of the homotrimers, FL_3 and ΔK_3. To clearly discriminate the these situations, a lysate of the cells co-expressing BmTorsoFL and BmTorsoΔK was fractionated by reducing Tris-Acetate SDS-PAGE, after treatment with the cross-linking reagent. As shown in Fig. S4A, by the BS^3 treatment, a new band appeared at 207 kDa, between the homo-oligomer bands of BmTorsoFL and BmTorsoΔK at 237 and 171 kDa, respectively. Since the same cells provided only the monomer bands of BmTorsoFL and BmTorsoΔK without the cross-linking treatment, at 105 and 72 kDa, respectively, the 207-kDa band was generated by hetero-oligomer formation between BmTorsoFL and BmTorsoΔK.

The molecular mass of the hetero-oligomer band (207 kDa) is approximately the average between those of the homo-oligomer bands (237 kDa and 171 kDa), and the 207-kDa band was the only band that newly appeared between the homo-oligomer bands. Therefore, these data were completely consistent with the simulated result of the dimer model in Fig. S4A. The oligomeric complex of Torso must be a *dimer*.

Without the cross-linking treatment, the truncated Torso mutant BmTorsoΔK ran as a dimer band at ~170 kDa (shown by the arrow D) on the non-reducing neutral-pH Bis-Tris gels, and an additional band of a higher-oligomer (the arrow H) appeared (Fig. S4B). Since the molecular mass of the higher-oligomer is estimated as ~340 kDa, it might be a tetramer. In the case of BmTorsoΔK, a monomer band was not observed, while the full-length BmTorsoFL ran as a faint monomer band on the same non-reducing gel (Fig. 4A). Probably because of the reduced steric hindrance in the intracellular region, the truncated mutant may form oligomers more easily, resulting in the formation of not only the dimer but also the unexpected tetramer. In comparison to the results in Fig. S4A, the BmTorsoΔK tetramer may be less effectively cross-linked by BS^3 than the dimer. The truncated mutant maintains the dimer formation ability.

4. Structural difference between the disulfide-bond-mediated dimer of the wild-type silkworm Torso and the non-covalently-associated dimer of its phenylalanine mutant

As proposed in Fig. 8, the non-covalently-associated dimer of the silkworm Torso phenylalanine mutant may adopt a different structure from that of the disulfide-bond-mediated dimer of the wild-type Torso. The structural difference between the dimers may cause a difference in their intracellular autophosphorylation sites. As a result, the phenylalanine mutant cannot elicit the ERK phosphorylation in the downstream signaling pathway, whereas the wild-type receptor facilitates it ligand-dependently.

To elucidate the structural difference between the dimers, we treated the receptors with two different cross-linking reagents (other than BS^3). As shown in Figs. 3A and 7B, both the wild-type and phenylalanine-mutant receptor dimers were efficiently trapped by the treatment with 0.3 mM BS^3, either with or without the PTTH stimulation. However, with another reagent possessing a longer spacer, Sulfo-EGS, the wild-type dimer was not trapped anymore, while the mutant dimer was apparently trapped by the cross-linker (Fig. S5). This difference in the reactivity to Sulfo-EGS clearly shows that a structural difference exists between the dimers.

Even though the non-covalently-associated phenylalanine mutant dimer was trapped with Sulfo-EGS possessing a longer spacer, this does not simply indicate that the distance between the two receptor molecules in the mutant dimer is longer than that in the wild-type dimer, because the mutant dimer was also more effectively trapped by BS^2G (also known as Sulfo-DSG), which has a shorter spacer than BS^3. The removal of the disulfide bridges in the transmembrane region may alter the spatial arrangement of the reactive free amino groups in the extracellular regions of the Torso dimer.

5. Primers used in this study

DNA primers used in this study are listed in Table S1.

6. References in this Supplementary Information

[S1] http://www.cbs.dtu.dk/services/SignalP/ (As of January 31, 2015)

[S2] http://www.cbs.dtu.dk/services/TMHMM/ (As of January 31, 2015)

[S3] Plotnikov, A. N., Schlessinger, J., Hubbard, S. R. & Mohammadi, M. Structural basis for FGF receptor dimerization and activation. *Cell* **98**, 641–650 (1990).

```
BmTorso     1:MYSEGKLLKVFL-I-------FAGFIIF-----SLCGGEVVS-QRYPPAPGLLKYLE-QDVCYSLYYYLNWTSLADCKTNFEETGIS-DV--PSTVKVRCQSKNSIRFETE---------P--SEHW-----------QLFILMEHDNFDPIPFTLIEPNNVFGELITTANKEYQIWSTYLDEYGT 145
DmTorso     1:MLIF--YAKYAF-I-------FWFFV------GSNQGGEMLLMDKISHDKTLLNVTACTQNCLEKGQMDFRSCLKDCRINGTFPGALRKVQENYQMNMICRTESEIVFQIDWVQHSRGTEPAPNATYIIRVDAVKDDNKETALYLSDDNFLILPGLESNSTHNITALAMHGDGSYSLIAK--DQ--T 166

DmPVR       1:MAML--PR---LILL-P---LLLILRI----SWSDA----VPLQQFSPDPDDSIENCGGENGAPLMTPCKSAI--------------ILDAQTSTTLKCEDDEPMSWWTSQSQYVHVKSFDNTEDPARPFGTSLH--LIEVTA--DYVAAYYCVKTSKFSQIAKEEQSDEAMIELVNQGYASSIYV 151
DmBTL       1:MA------KV-PITL------VMIIAIV-----S-A-----------AADLGCDYGHHRCYIDVTVENSPRQRHLLS------------DM-D-ITLQCVRPMAKWFYEDKFQLRATLLRLERAQS------------------GNSGNYGCLDSQNR--WYNISLVVGHKEPVGND-IASFVKL 120
DmHTL       1:MAAAWSWRASHSTITMTSGSLVVLFLLLSIWQPAVQ---------------------------------------------------------------------------------------------------------------------------------------- 36
hPDGFRA     1:MGTS-HPA---FLVL-----GCLLTGL----SLILC----------------------------QLSLPSILPNEN---------EHIMQAGQTLHLQCRGEAAHKWSLPEMVSKESERLSITKSACGRNGKQFCSVLEVSSASAAHTGLYTCYYNHTQTEENELEGRHIYIYVPDPD-------- 125
hVEGFR1     1:MV---SYWDTGVLL------CALLSCLL-LTGSSSG----------------------------SKLKDPELSLKGT---------QHIMQAGQTLHLQCRGEAAHKWSLPEMVSKESERLSITKSACGRNGKQFCSTLTLNTAQANHTGFYSCKYLAVPT-SKKKET-----E-S-AIYIFISDTG 132
hFGFR1      1:M---WSW-KC----------LLFWAVLVT-ATLCTA----------------------------RPS-PTLPEQAQPWGAPVEVESFLVHPGDLLQLRCRLRDDVQSINWLRDGVQLAESNRTRIT--------GEEVEVQDSVPADSGLYACVTSS-PSGSD---TTYFSVNVSDALPSSEDDDD 130

BmTorso   146:LQDWMEGPIVLKFDQRNQQPDDIKYNVTQEFKYIILGNDSYTINGKFVWNTTGDRDLC-FDIANICQNTNMKHAKI---WPTAH--PSFDVENLVLNDECEIHVKGIHGTTK-HKYKT- 257
DmTorso   167:FATLI----------RGYQPS--KMGAVNLLRFVPQPDDLHHIAAEIEWKPSAESN-CYFDMVSYSTNSVNMDEPLEVQFRDRKKLYRHTVDNLEFDKQYHVGVRTVNIMNRLESDLQW 272

DmPVR     152:YVN------DPD-TK--------LVDS-HNV----VTARQYTDVVIPCKPAMPDTEVLLETSNGEMHSS--KSVGRYDPQRGFTIEIRSIV--DGGDYYCRPNPPFPHNEEEMTSIEVR 246
DmBTL     121:EDAPALPESDLFFQPLNESRSLKLLQPLPKT----VQRTAGGLFQLNCSPMDPDAKGVNISWLHNDTQILGG-RGRIKLKRWSLTVGQLQ-PEDAGSYHCLCVEQDCQRSNPTQLEVIE 234
DmHTL        ----------------------------------------------------------------------------------------------------------------------
hPDGFRA   126:--------------------VA-FV--PLGMTDYLVIVEDDDSAIIPCRTT-DPETPVTLHNSEGVVPASYDSRQGFNG--TF-----TV-----GPYICEATV-KGKKFQTIPFNVYA 207
hVEGFR1   133:--------R-P--FVEMYSEIPEIIHMTEG-----------RELVIPCRVT-SPNITVTLKKFPLDTLIPDGKRIIWDSRKGFIISNATY--KEIGLLTCEATV-NGHLYKT---N--- 219
hFEGR1    131:DDDSSSEEKETDNTKPNRMPVAPYWTSPEKMEKKLHAVPAAKTVKFKCPSSGTPNPTLRWLKNGKEFKPDHRIGGYKVRYATWSIIMDSVVPSDKGNYTCIVENEYGSINHTYQLDVVE 249

BmTorso   258:-----PSCFE-LP-E---CFLNNMEPEIPQDVAIAADQDLRGWWNINVAWAKPHFQPEIYNVTVRANMIRSIILPGNATETTFRNIPNTFL-----SAGKIYNVSVYAIIGQKASHTSRRAFTP-----GMLR--- 369
DmTorso   273:LPIAVPSCLDWYPYNYTLCP-----PHKPENLTVTQKQYLPNILALNITWARPRYLPDNYTLHIFDLFKG-----GTELNYTLDQNRSHFYVPKITVLGSHFEVHLVA---QSAGGKNVSGLTLDKVHRGVLLSEG 395

DmPVR     247:FIGNGHIDKDGKPLPKPVIRSSV--EHH---VF-TDTNFTLDCEQSAYVESVYGMEWFTPSRDENRIFASQSRTD--PKTRN-STHQTGRSTLTVL---NAQPSDTGLYKCVTTDNSNQNVQRATYRIKVLKQNES 370
DmBTL     235:----------SRKHTVPMLKPGYPRNTS---IAL-GDNVSIECLLEDSALEPKITWLHKGNADNIDDLLQRLREQSQLPVDVTRLITR-MDEPQVLRLGNVLMEDGGWYICIAENQVGRTV-AASYVDLYSPSDTT 354
DmHTL      37:---------------------------------------------------------------------------------------------------------------VEGRRQMANSQEMIKDHLGAR 57
hPDGFRA   208:----------LKATSEL--DLEMEALKT---VYKSGETIVVTCAVFNNEVVDLQWTYPGEVKGKGITMLEE-IKVPSIKLVYTLT---------VP---EATVKDSGDYECAARQATREVKEMKKVTISVH--EK- 312
hVEGFR1   220:-----------YLTHRQTNTIIDVQISTPRPVLLRGHTLVLNCTATTPLNTRVQMTWSYPDEKNKRASVRR-RIDQSNSHANIF--------YSVLTIDKMQNKDKGLYTCRVRSGPSFKSVNTSVHIYDK----- 330
hFGFR1    250:-----------RSPHRPILQAGLPANKT---VAL-GSNVEFMCKVYSDPQPHIQWLKHIEVNGSKIGPDNL-PYVQILKTAGVNTTDKEM---EVLHLRNVSFEDAGEYTCLAGNSIGLSHHSAWLTVLEALEERP 366

BmTorso      --------------------------------------------------------------------------------------------------------------
DmTorso      --------------------------------------------------------------------------------------------------------------

DmPVR     371:YLNVGEPSGHYNVQEYANRTIQMTANFEGFPTPSFSWFKPDGTEVRQSENNFKILST-ELSTMLQVLNAQLQDSGTYVLRGSN-SFGVVQREYNVSVM-- 466
DmBTL     355:TVRTTTTT-VASPIPTASTGEDNDDDVENPAAEASG-GVGPP-VFRKE--KR----------LQHS---------------------------------- 405
DmHTL      58:SQNKTPAITNNANQ------SSTSSADLDDGAADDDDNKADLP-VNVSS--KPYWRNPKKMSFLQTR--------------------------------- 115
hPDGFRA   313:--------GFIEIKPTFSQ-LEAVNLHEVKHFVVEVRAYPPPRISWLKNNLTLIENLTEITTDVEKIQEIRYRSKLKLIRAKEEDSGHYTIVAQNEDAVK 403
hVEGFR1   331:--------AFITVKHRKQQVLETVAGKRSYRLSMKVKAFPSPEVVWLKDGLPATEKSARYLTRGYSLIIKDVTEEDAGNYTILLSIKQSNVFKNLTATL- 421
hFGFR1    367:AVMTSPLY-L-E------------------------------------------------------------------------------------------ 376

BmTorso      ------------------------------------------------------------------------------------------------------------------------------------------------------------------
DmTorso      ------------------------------------------------------------------------------------------------------------------------------------------------------------------

DmPVR     467:-----DAPALKMSD-----------AYVQVGS--VARLECTVRSYPPAIVTFFFRPCSLEPQWPTCSVLNQNFSLPSEQEKYQFQTRPRPGKLSVERIYE--------VSFLPTEPGILTCIAQNIIDGKERRTLTKAHVLLGNISEN 588
DmBTL     406:-----------LS-----------------GN--TVNLACPVYGKANITWTKDKKPLNRELGVYVQKNWTLRFVEATSE---DSGL------------------------YNCKVCNAWGCIQFDFSVQINDRTRSAPIIVV------ 490
DmHTL     116:-----------PS-----------------GS--LLTLNCHALGNPEPNITWYRNGTVDWTRGYGSLKRNRWTLTMEDLVPGDCGN------------------------YTCKVCNSLGCIRHDTQVIVSDRVNHKPILMTG----- 204
hPDFGRA   404:SYTFELLTQV-PSSILDLVDD-------HHGSTGGQTVRCTAEGTPLPDIEWM--IC-KDIK--KC----NNETSWTILANNVSNIITEIHSRDRSTVEGRVTFAK--V-EET-IA--VRCLAKNLLGAENRELKLVAPTLRSELTVA 528
hVEGFR1   422:-----IV-NVKPQIYEKAVSSFPDPALYPLGSR--QILTCTAYGIPQPTIKWFWHPCNHNHSEARCDFCSNNEESFILDADSNMGNRIESITQRMAIIEGKNKMASTLVVADSRISGIYICIASNKVGTVGRNISFY-------ITD- 553
hFGFR1       ------------------------------------------------------------------------------------------------------------------------------------------------------------------

BmTorso      ----------------------------------------------------------------------------------------------------
DmTorso      ----------------------------------------------------------------------------------------------------

DmPVR     589:MTIYGF--DKDHKIAKEDNVNFTCEALAYHFDGNLKWF---INGEDLKESDSVHIETSHTKYSYKSTVHITTISDRDRGTYECRAYHNDKDAAYSSR-EIDLYVHDP 689
DmBTL     491:--------PQNQTVKVNGSLVMKCTVYSDLHPTVSWKRVVLKNASLDGLKSVEIQNLNFTVTNDSVVLTLRNVTFDQEGWYTCLASSGLGRSNSSVYLRVVSPLPPL 589
DmHTL     205:--------PLNLTLVVNSTGSMHCKYLSDLTSKKAWIFVPCHGMTNCSNNRSII-------AEDKDQLDFVNVRMEQEGWYTCVESNSLGQSNSTAYLRVVRSLHVL 296
hPDGFRA      ----------------------------------------------------------------------------------------------------
hVEGFR1   554:VP-NGFHVNLEKMPTEGEDLKLSCTVNKFLYRDVTWILLRTVNNRTMHYSISKQKMAITKEHSITLNLTIMNVSLQDSGTYACRARNVYTGEEILQKKEIT--IRDQ 657
hFGFR1       ----------------------------------------------------------------------------------------------------

BmTorso      ----------------------------------------------------------------------------------------------------
DmTorso      ----------------------------------------------------------------------------------------------------

DmPVR     690:SAPQWTNGGQEGHS-KIKRKLSQTLELECASTAVPVAIVRWFKDDKEVTESKLRHIIEKESK-LITHLYPGDEGVYKCVVENRLDRIERS-F-TVVISDLPGIS-MA 792
DmBTL     590:EI-YALLHAHPLG--------------------------------------------------------------------------------------------- 601
DmHTL     297:EAGVASGSLHSTS--------------------------------------------------------------------------------------------- 309
hPDGFRA      ----------------------------------------------------------------------------------------------------
hVEGFR1   658:EAPYLLRNLSD-HTVAISSSTT----LDCHANGVPEPQITWFKNNHKIQQEPGIILGPGSSTLFIERVTEEDEGVYHCKATNQKGSVESSAYLT-V-QGTSDKSNLE 758
hFGFR1       ----------------------------------------------------------------------------------------------------
```

Figure S1. | Alignment of the extracellular amino-acid sequences of silkworm and fruit-fly Torso with those of typical RTK subclass-III members. All the sequences are from the UniProt Knowledge Base, although a slightly different sequence is encoded by the BmTorso gene cloned in this study.

Sulfo-DST (Di[sulfosuccinimidyl] tartrate)

spacer length: 6.4 Å

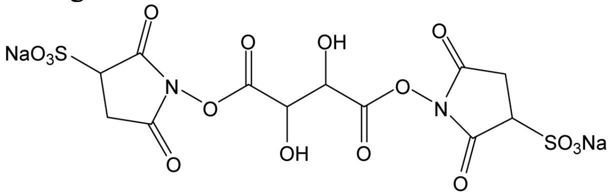

BS[3] (Sulfo-DSS) (Di[sulfosuccinimidyl] suberate)

spacer length: 11.4 Å

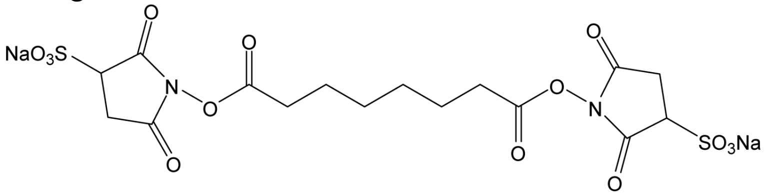

Sulfo-BSOCOES (Bis[2-(sulfosuccinimidyloxycarbonyl)-ethyl]sulfone)

spacer length: 13.4 Å

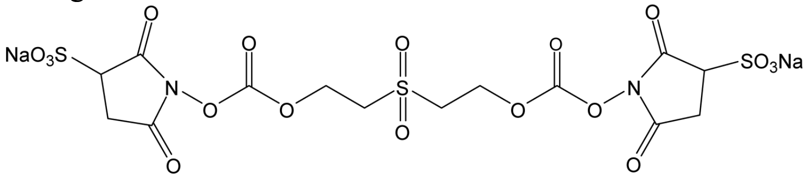

Sulfo-EGS (Ethyleneglycol bis[sulfosuccinimidylsuccinate])

spacer length: 16.1 Å

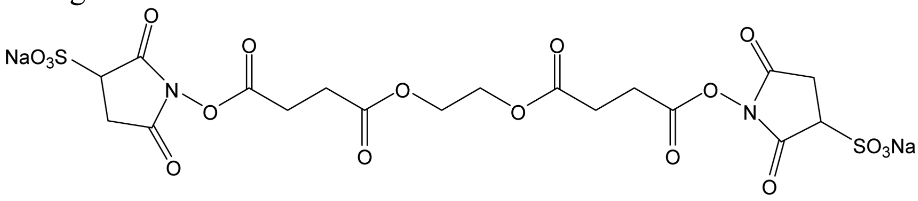

Figure S2. | Chemical structures of the homo-bifunctional sulfo-succinimidyl esters used in this study.

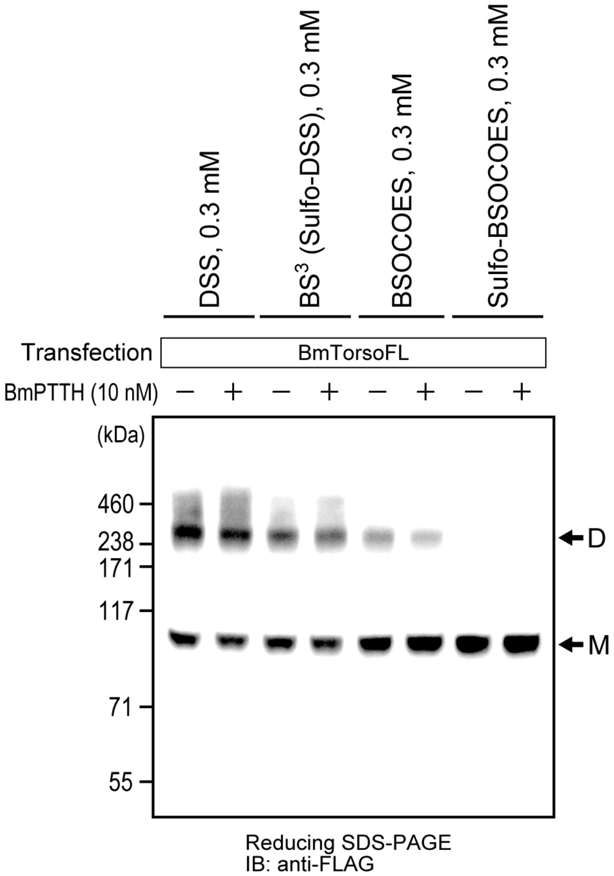

Figure S3. | Detection of Torso oligomerization, using the hydrophobic cross-linking reagents, DSS and BSOCOES, and their sulfonated derivatives, BS^3 (Sulfo-DSS) and Sulfo-BSOCOES, respectively.

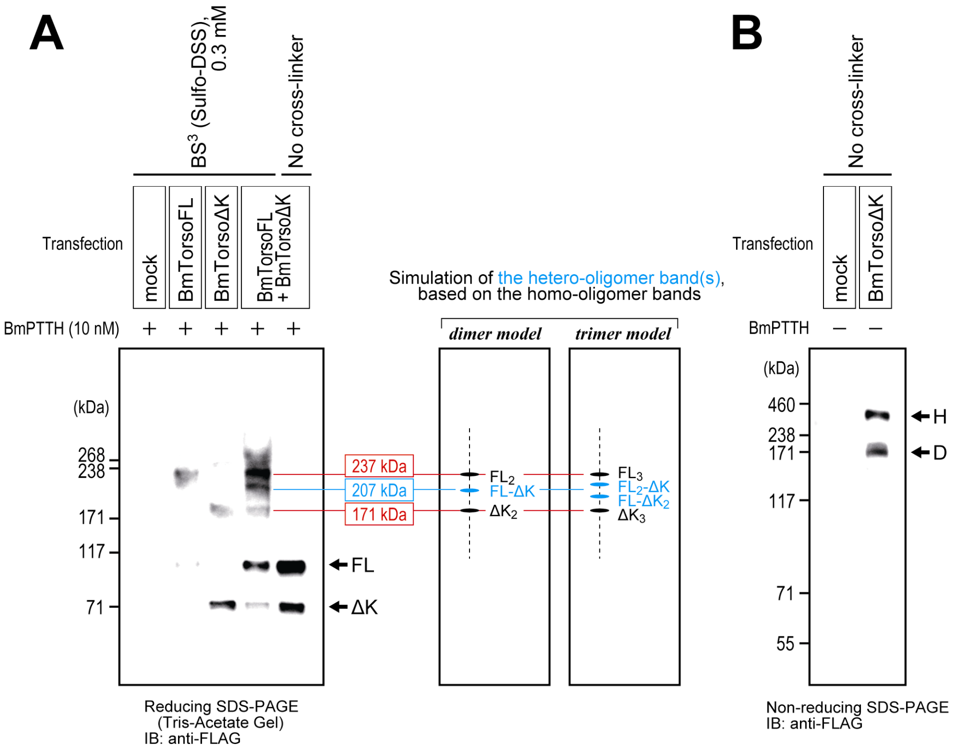

Figure S4. | Oligomerization of a truncated mutant of silkworm Torso (BmTorsoΔK), which lacks the C-terminal half of the intracellular kinase and its following tail. (A) Hetero-oligomerization with full-length silkworm Torso (BmTorsoFL) was detected by reducing Tris-Acetate SDS-PAGE, with the BS^3 treatment. (B) Homo-oligomerization was detected by non-reducing Bis-Tris SDS-PAGE, without the cross-linking treatment.

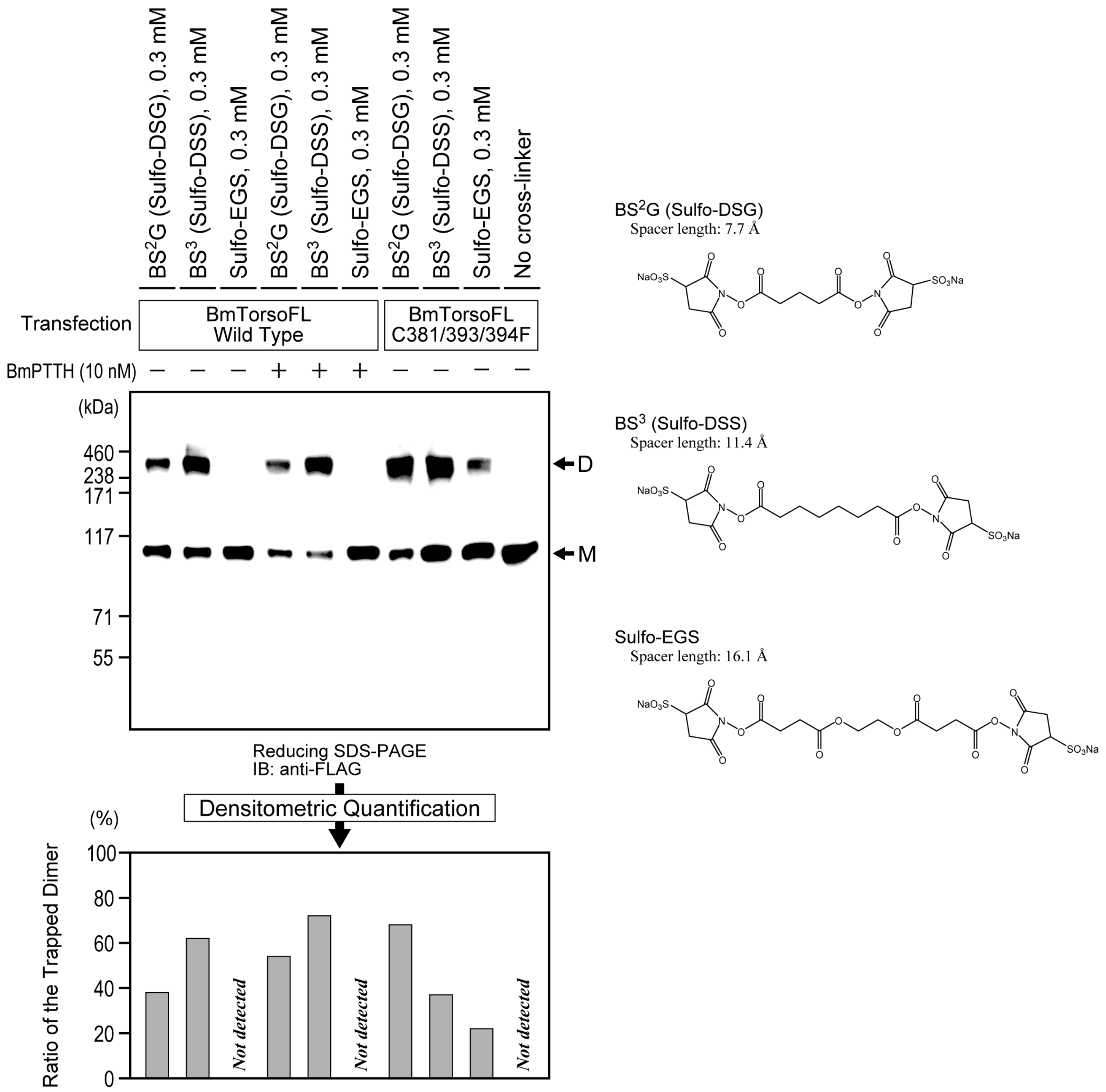

Figure S5. | Different reactivities to cross-linking reagents between the disulfide-bond-mediated dimer of the wild-type silkworm Torso and the non-covalently-associated dimer of its phenylalanine mutant. The wild-type dimer was not trapped by 0.3 mM Sulfo-EGS, either with or without the PTTH stimulation, while the phenylalanine-mutant dimer was apparently trapped by the cross-linker.

Table S1. Sequences of the primers used in this study

Experiment	Sequences
BmTorso cloning	
Degenerate F	ATGTATTCGGAAGGTAAA
Degenerate R	TCACGCGGTTTTAGTTT
DmERK cloning	
Degenerate F	ATGGAGGAATTTAATTCG
Degenerate R	CTTAAGGCGCATTGTC
pIZT-BmTorsoFL	
Construct F-*Spe*I	TTTACTAGTATGTATTCGGAAGGTAAA
Construct R-FLAG	AAACTCGAGTCACTTGTCATCGTCATCCTTGTAGTCGCCGCCCGCGGTTTTAGTTTGT
pIZT-BmTorsoΔK	
Construct F-*Spe*I	TTTACTAGTATGTATTCGGAAGGTAAA
Construct R-Δ590–807	AAACTCGAGTCACTTGTCATCGTCATCCTTGTAGTCACGACAGAAAGAGAGTA
pMT-BmTorsoEC	
Construct F-*Spe*I	TTTACTAGTATGTATTCGGAAGGTAAA
Construct R-EC-FLAG	AAACTCGAGTCACTTGTCATCGTCATCCTTGTAGTCGCCTGGAGTAAAAGCT
pIZT-BmTorsoFL (C381/393/394A)	
Construct F-OplE2	CGCAACGATCTGGTAAACAC
Construct R-OplE2	GACAATACAAACTAAGATTTAGTCAG
Mutation F-C393/394A	CTGCTGGCCGCAGGACACCGCCGCGCT
Mutation R-C393/394A	TCCTGCGGCCAGCAGGGTGGCCGC
Mutation R-C381A	GCGGGGGCAGCGGCGGGGGGGCTG
Mutation F-C381A	CGCCGCTGCCCCCGCGCCTGCCGT
pIZT-BmTorsoFL (C381/393/394F)	
Construct F-OplE2	CGCAACGATCTGGTAAACAC
Construct R-OplE2	GACAATACAAACTAAGATTTAGTCAG
Mutation F-C393/394F	CTGCTGTTCTTCGGACACCGCCGCGCT
Mutation R-C393/394F	TGTCCGAAGAACAGCAGGGTGGCCGC
Mutation R-C381F	GCGGGGTTCGCGGCGGGGGGGCTG
Mutation F-C381F	CGCCGCGAACCCCGCGCCTGCCGT
pIZT-DmERK	
Construct F-*Sac*	TTTGAGCTCATGGAGGAATTTAATTCG
Construct R-c-Myc	CCCTCTAGATCACAGATCCTCTTCTGAGATGAGTTTTTGTTCGCCGCCAGGCGCATTGTCTGG
