# Supplementary material for: Ligand-dependent responses of the silkworm prothoracicotropic hormone receptor, Torso, are maintained by unusual intermolecular disulfide bridges in the transmembrane region
Source: Sci Rep. 2016 Mar 1;6:22437. doi: 10.1038/srep22437 (PMC4772477; doi:10.1038/srep22437)
Supplement: Supplementary Information [file srep22437-s2.pdf]

Supplementary Information

# – Original Images of the Immunoblotting –

Ligand-dependent responses of the silkworm prothoracicotropic hormone receptor, Torso, are maintained by unusual intermolecular disulfide bridges in the transmembrane region

Tadafumi Konogami, Yiwen Yang, Mari H. Ogihara, Juri Hikiba, Hiroshi Kataoka & Kazuki Saito

All of the visualized bands in our immunoblotting data were recorded with BIO-RAD ChemDoc XRS+ instruments, using the “Blot > Chem Hi Resolution” mode. After the absence of signal saturation was confirmed by the Image Lab Software version 4.1, the images were directly saved as TIFF files, without any manipulation. In addition, to record the pre-stained molecular-mass marker bands on the blotted membranes, pictures of the same images were taken, using the “Protein Gel > Flamingo” mode, and they were also saved as TIFF files.

This Supplementary Information includes the original images of all immunoblotting results, before trimming for figure preparation. The trimmed areas in the images are shown by red boxes. For all of the immunoblotting data, two types of images are provided:

(a) An immunoblotting image acquired by the “Blot > Chem Hi Resolution” mode, and

(b) A pre-stained molecular-mass marker image acquired by the “Protein Gel > Flamingo” mode.

(a)

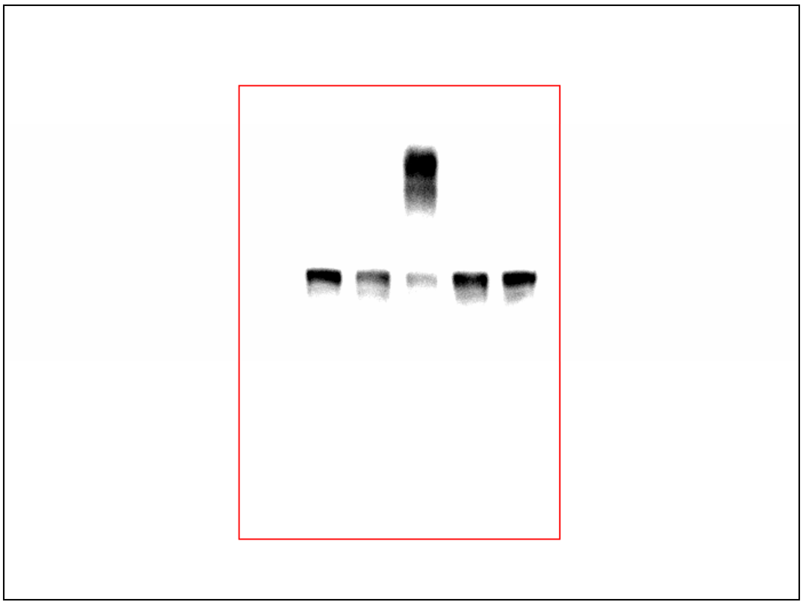

(b)

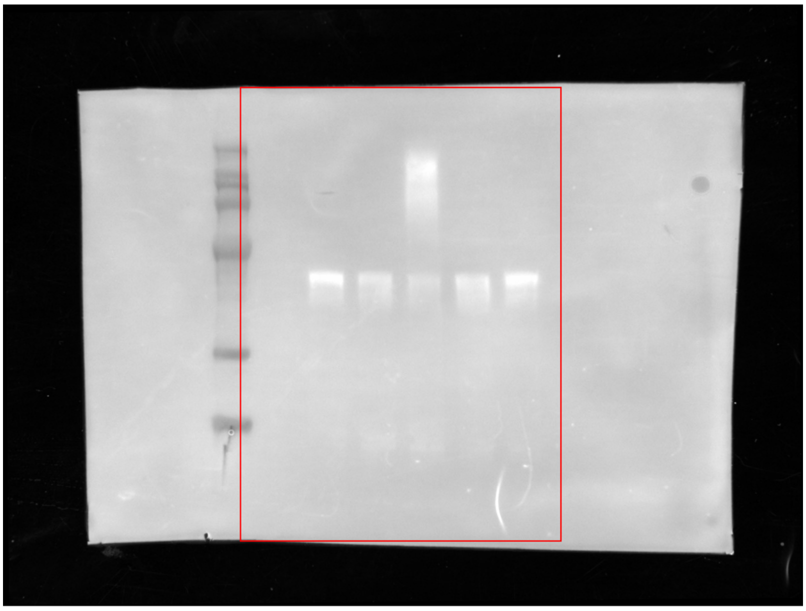

Original images for Fig. 2A

(a)

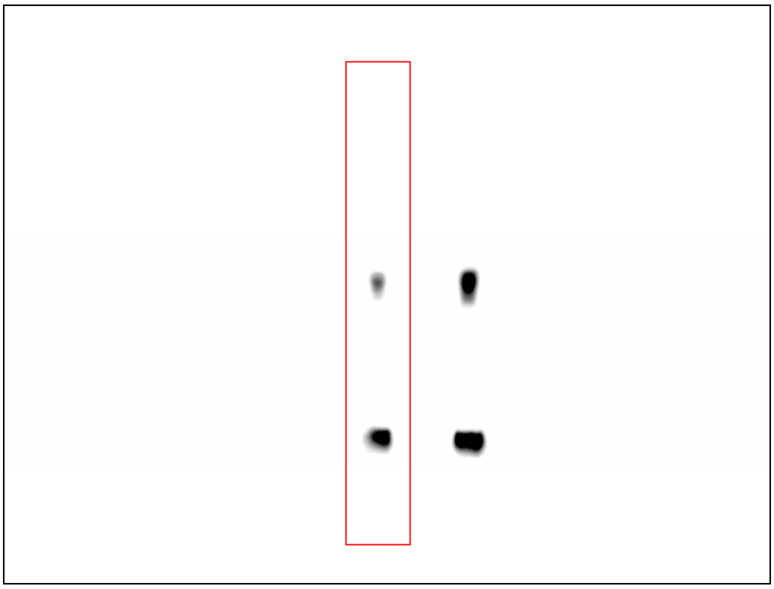

(b)

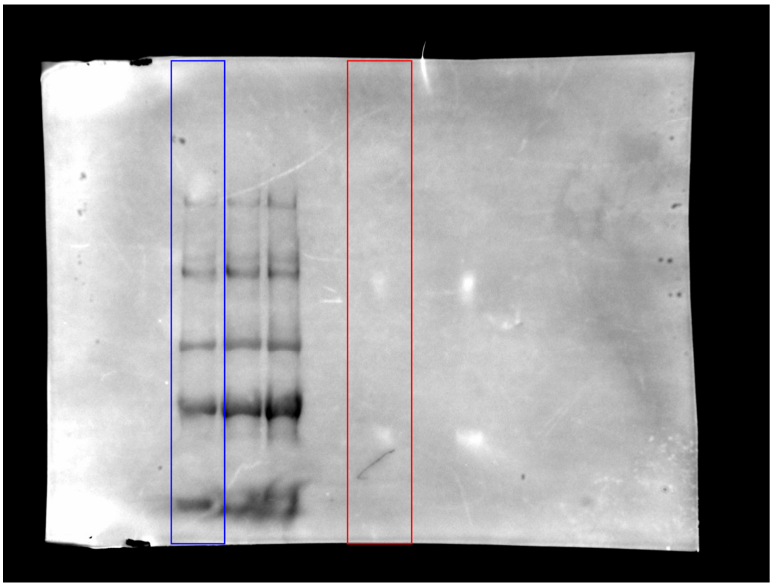

Original images for Fig. 2B

(a)

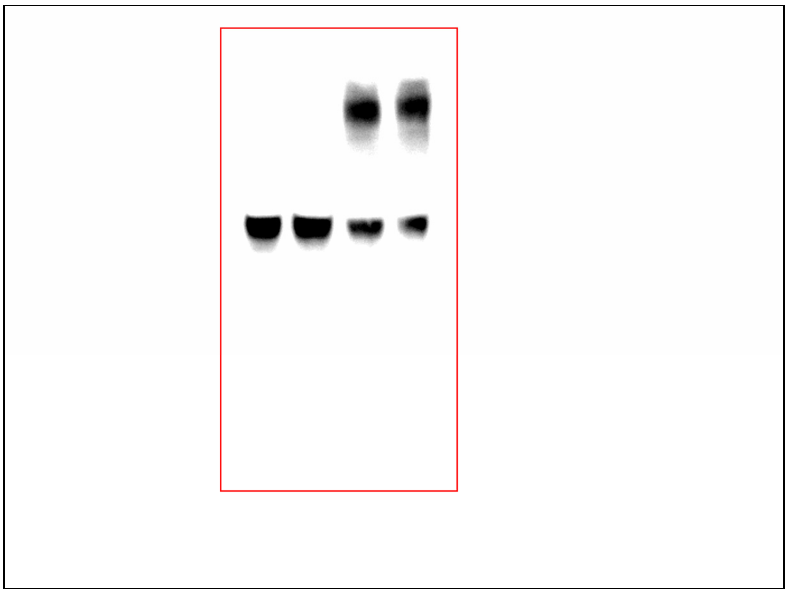

(b)

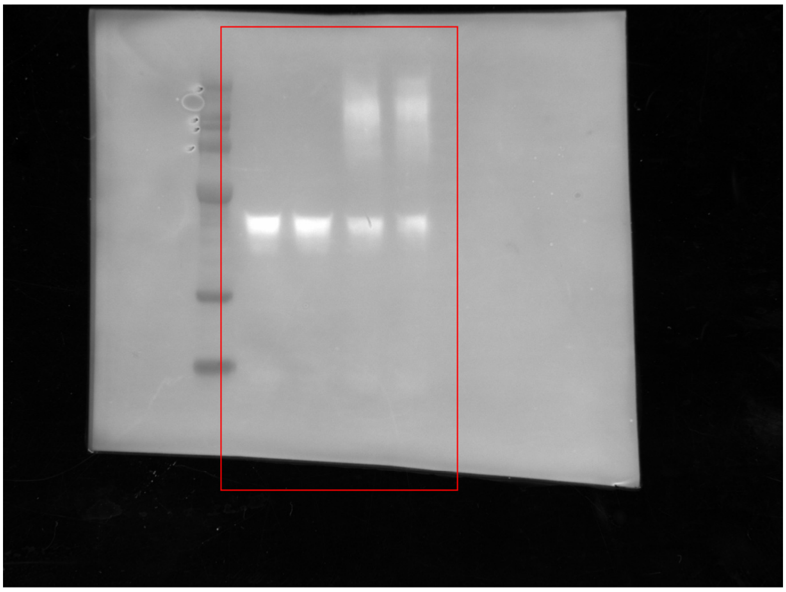

Original images for Fig. 3A

(a)

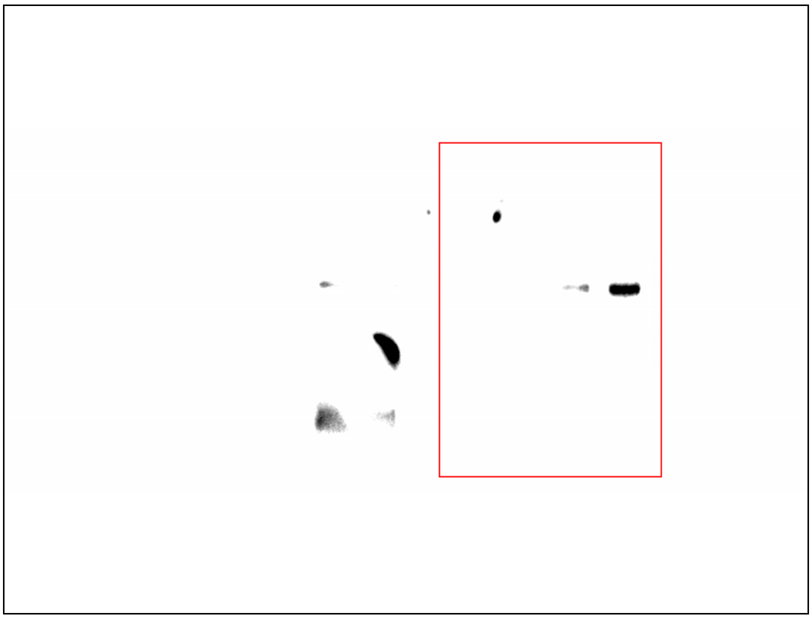

(b)

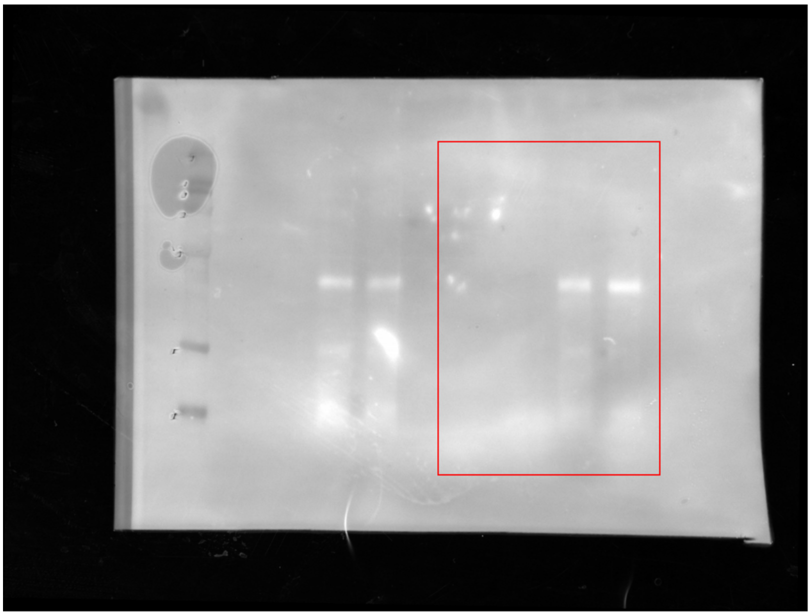

Original images for Fig. 3B (pTorso)

(a)

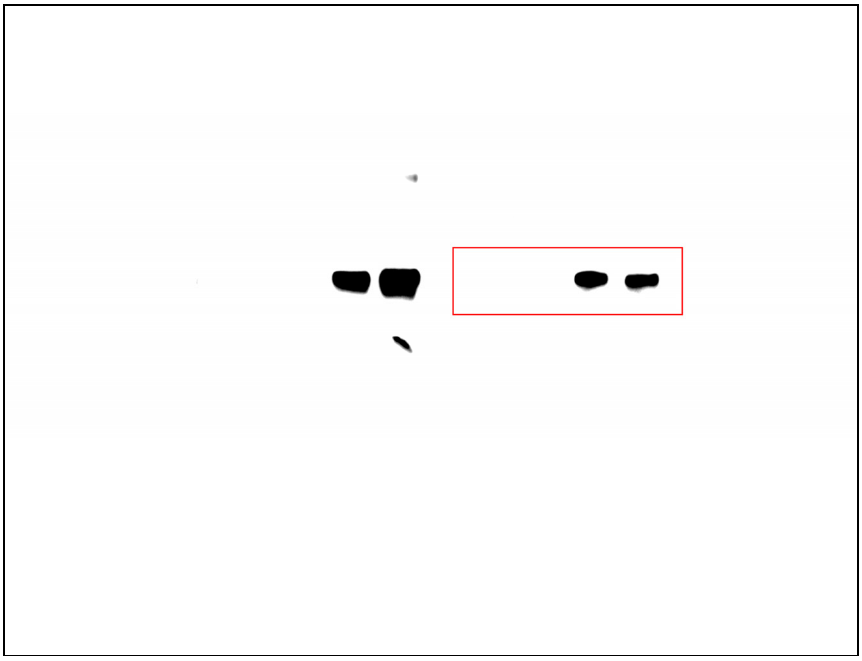

(b)

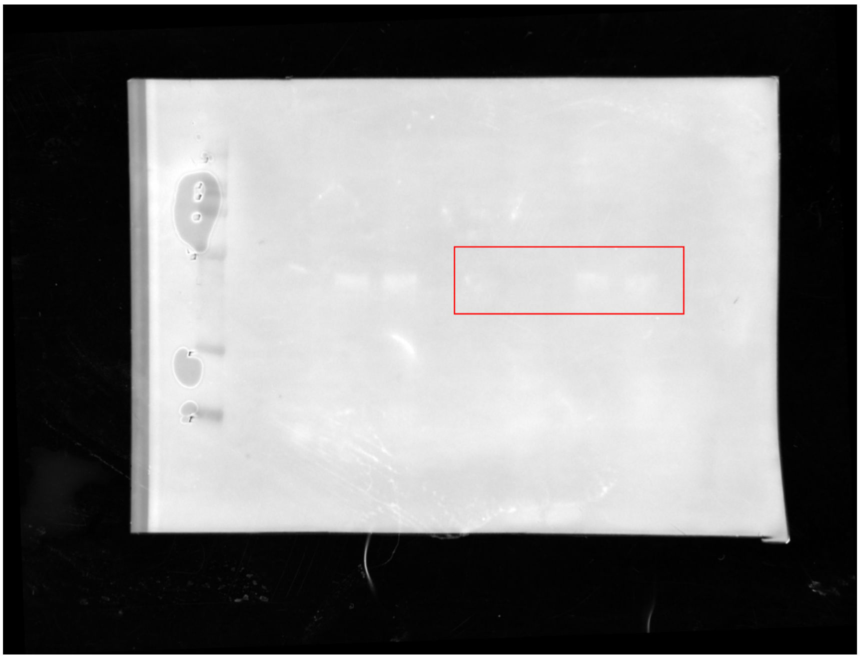

Original images for Fig. 3B (Reprobe)

(a)

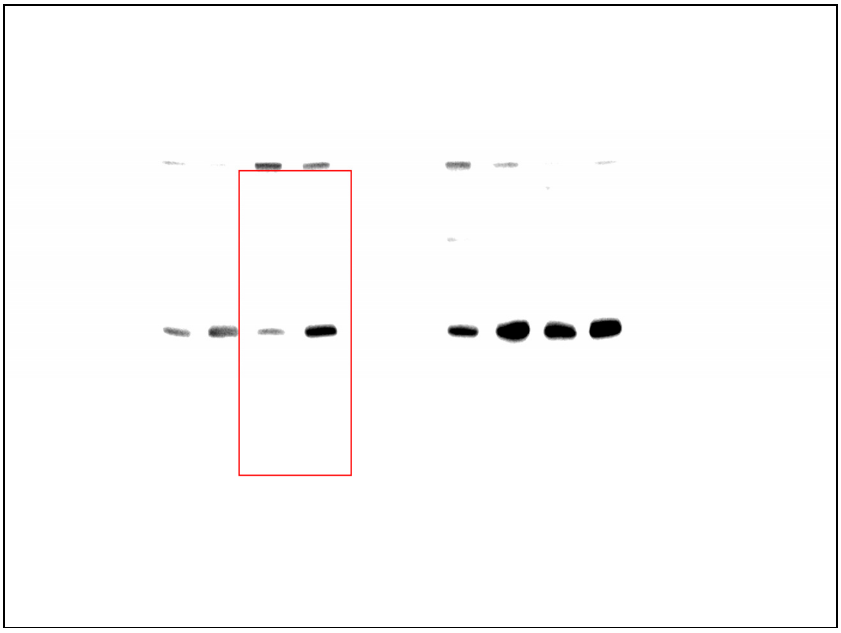

(b)

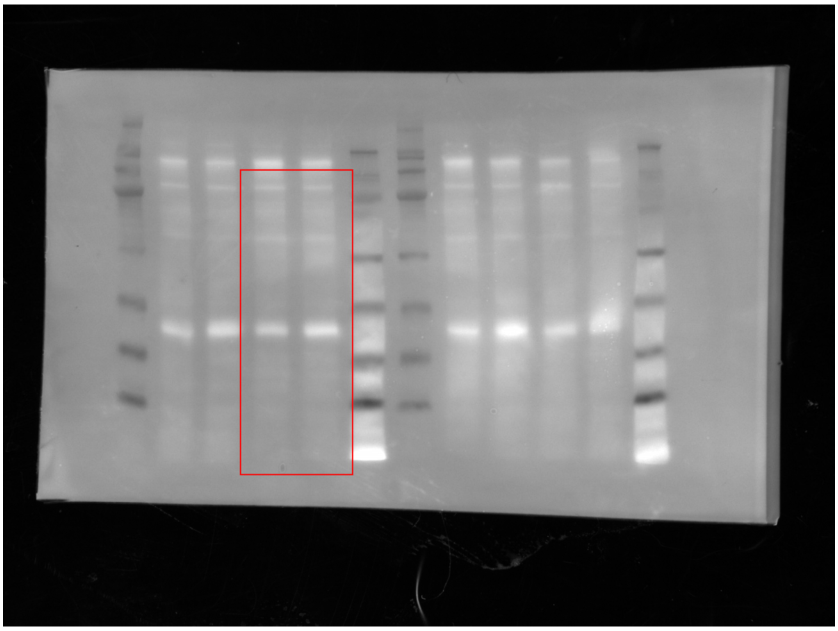

Original images for Fig. 3C (pERK)

(a)

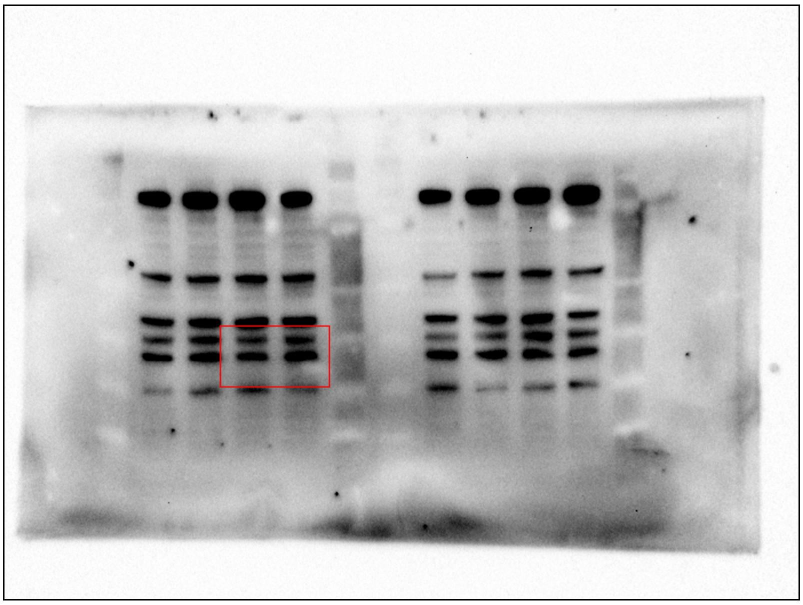

(b)

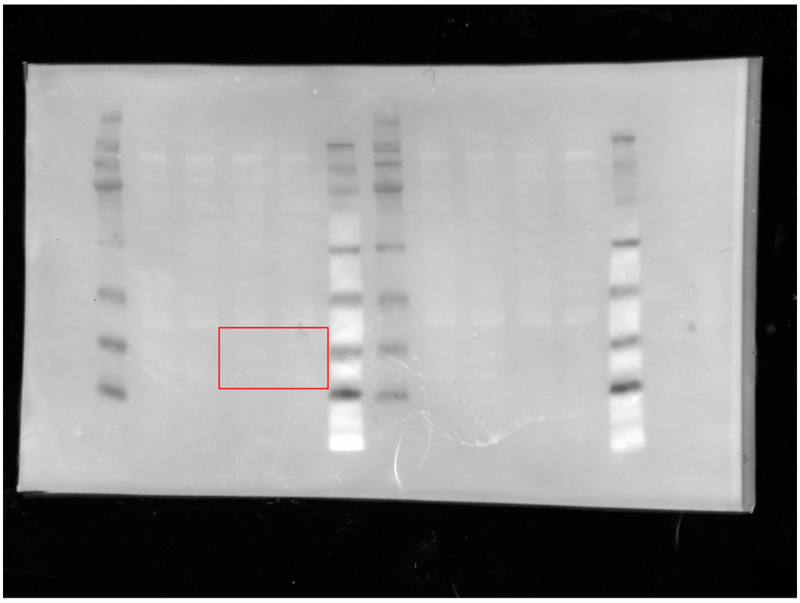

Original images for Fig. 3C (Reprobe)

(a)

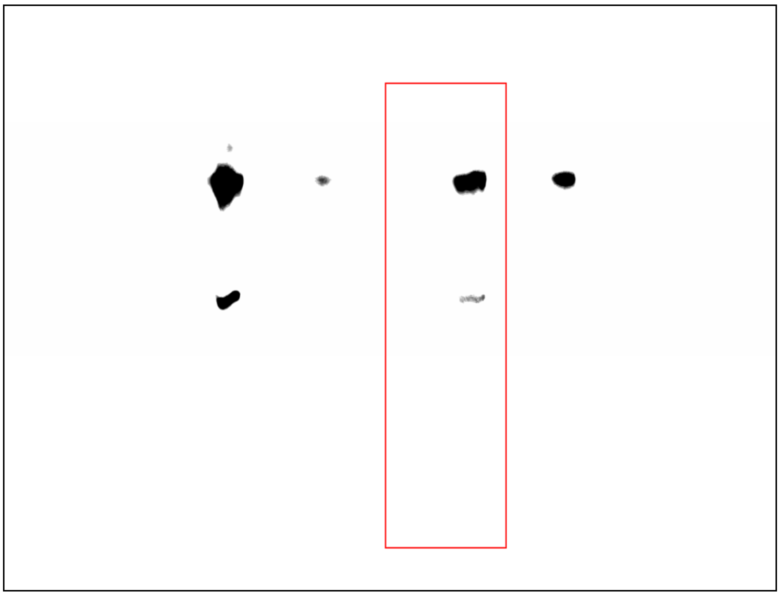

(b)

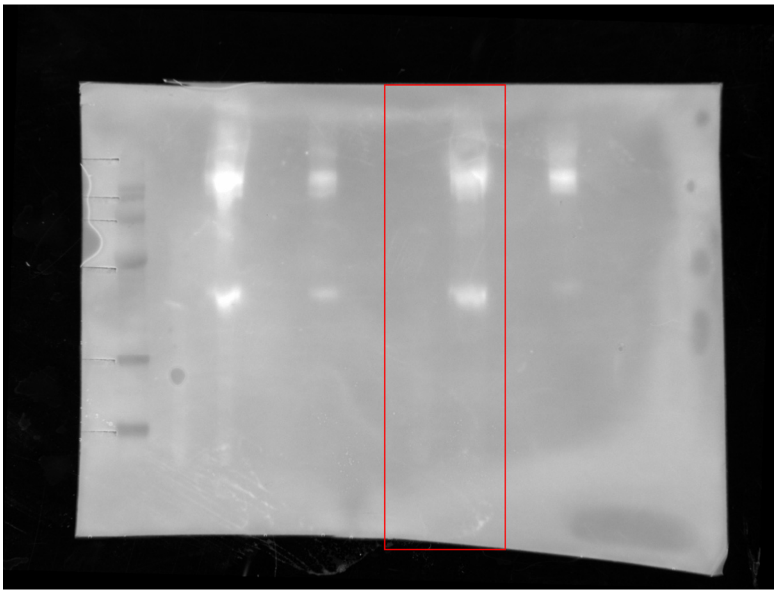

Original images for Fig. 4A

(a)

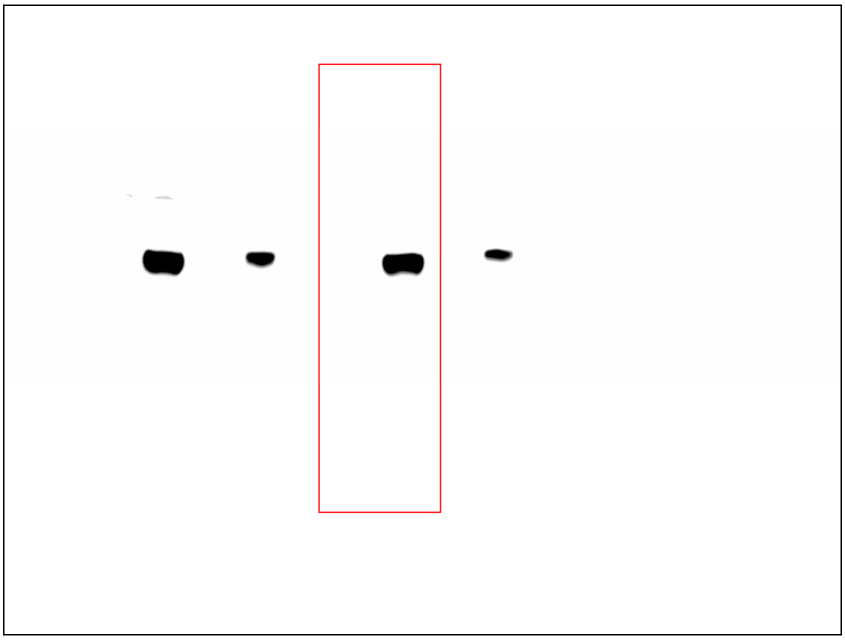

(b)

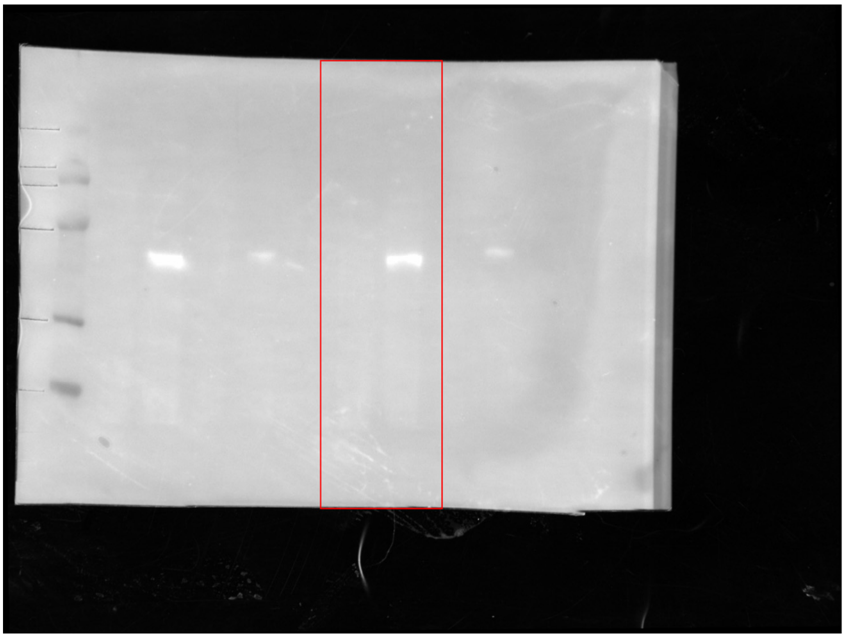

Original images for Fig. 4B

(a)

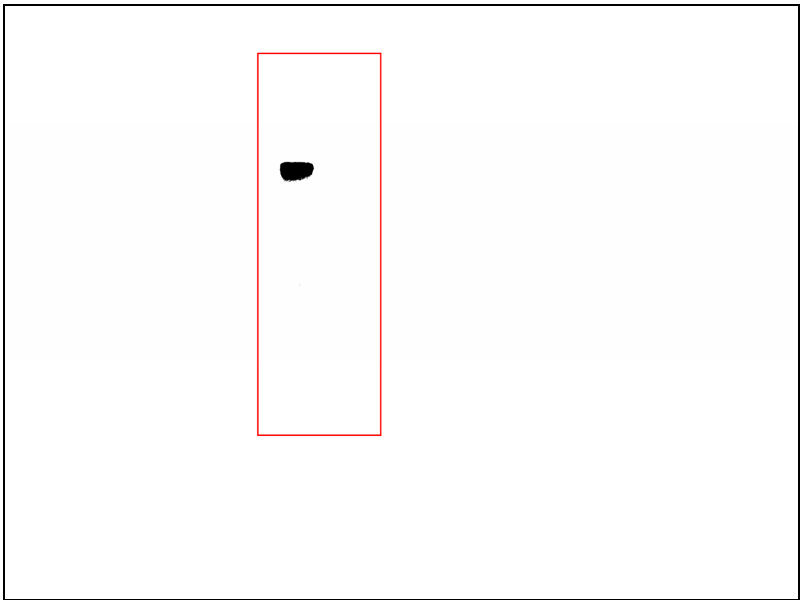

(b)

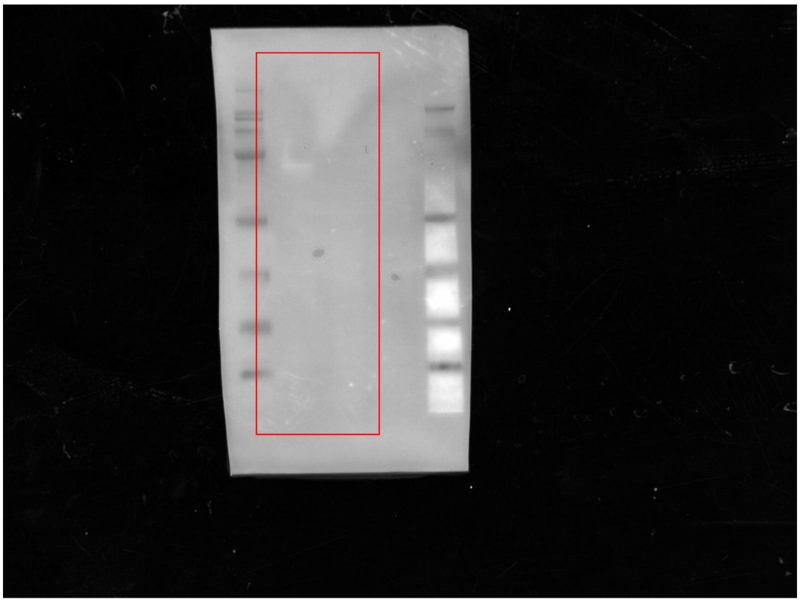

Original images for Fig. 5A (Cell Lysate)

(a)

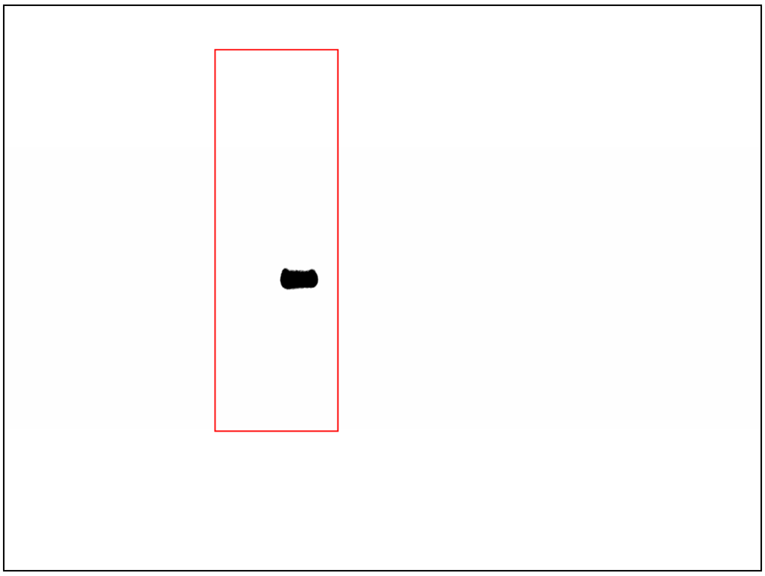

(b)

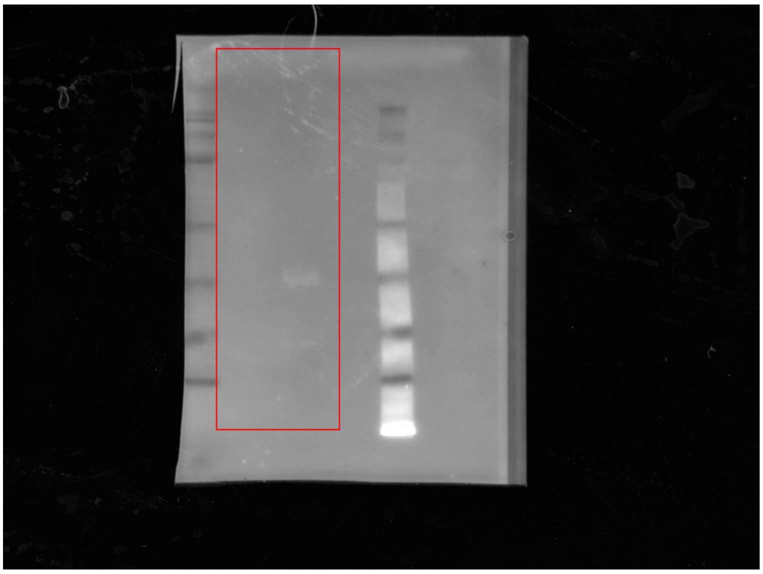

Original images for Fig. 5A (Culture Medium)

(a)

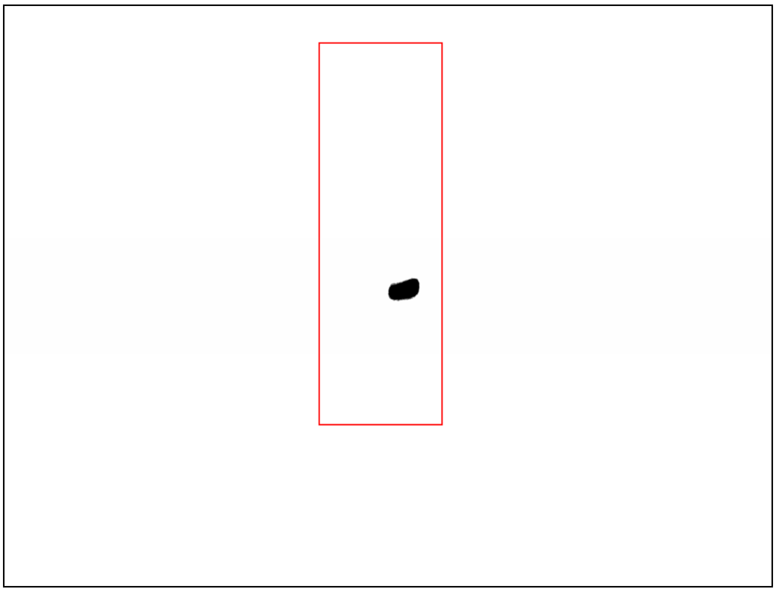

(b)

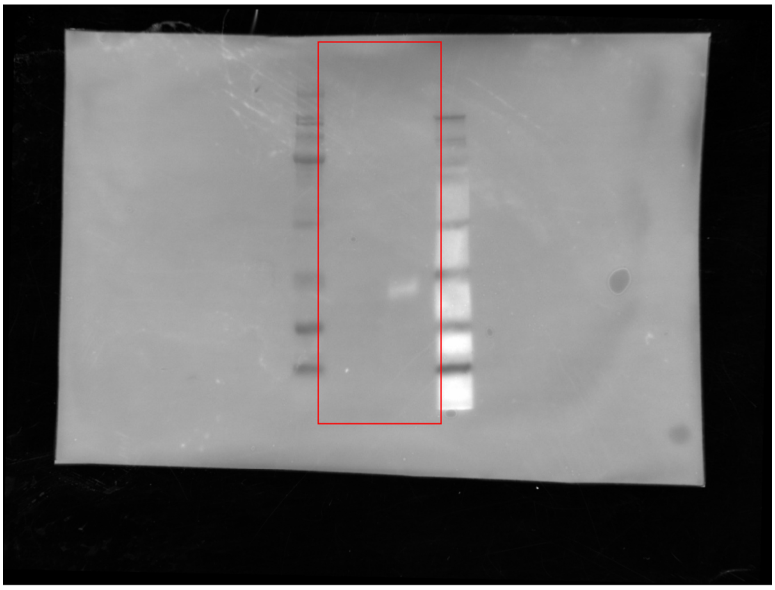

Original images for Fig. 5B

(a)

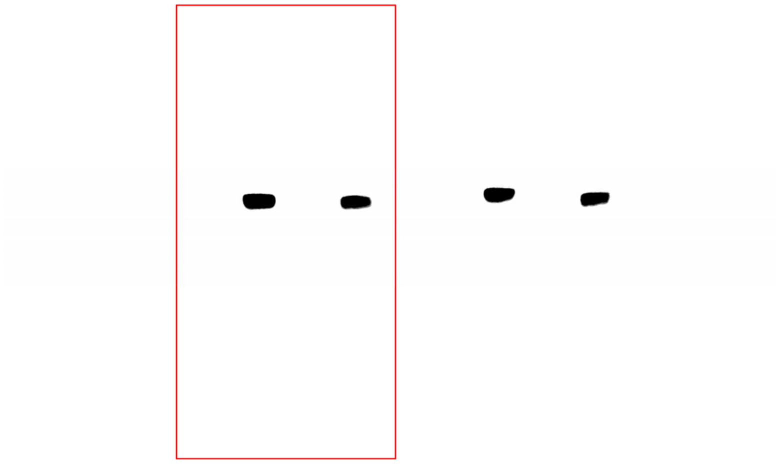

(b)

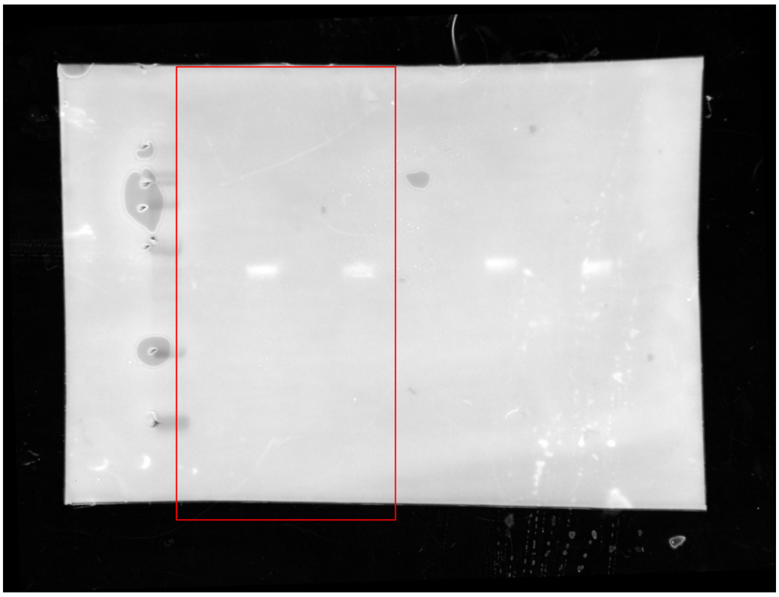

Original images for Fig. 6A

(a)

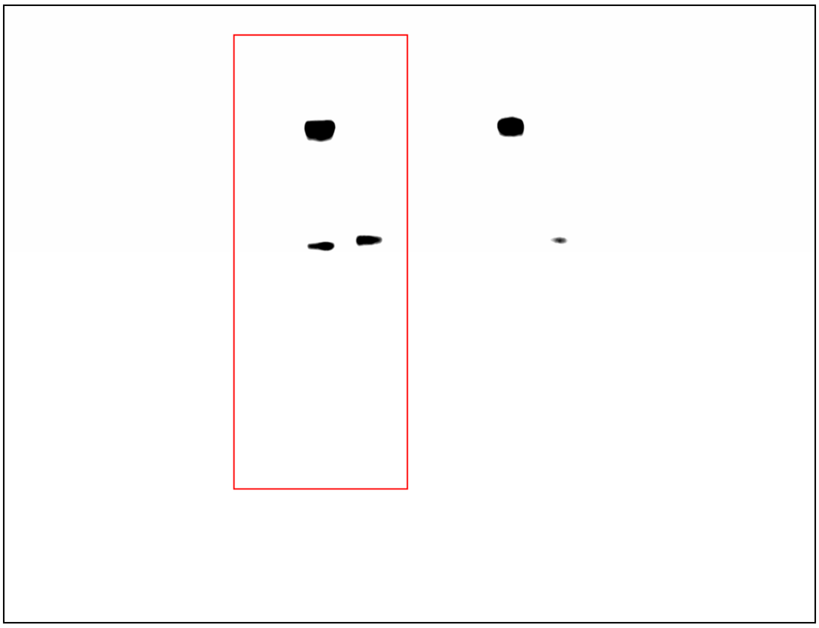

(b)

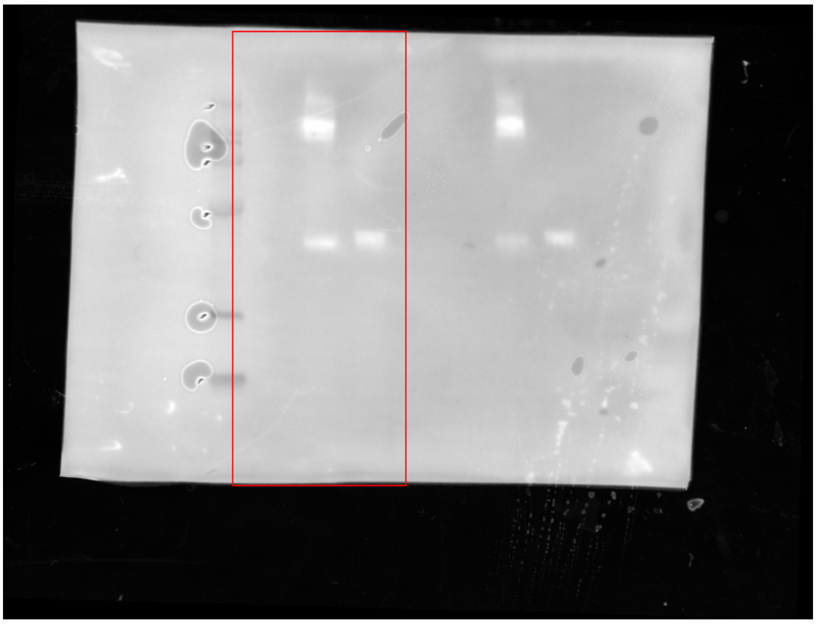

Original images for Fig. 6B

(a)

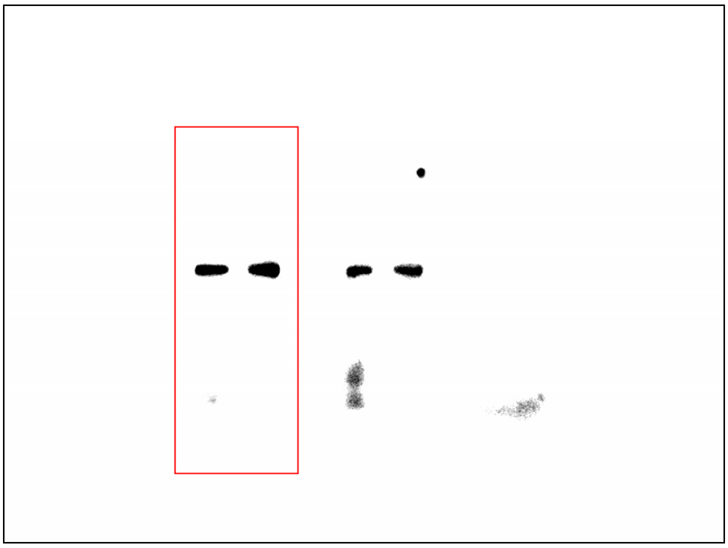

(b)

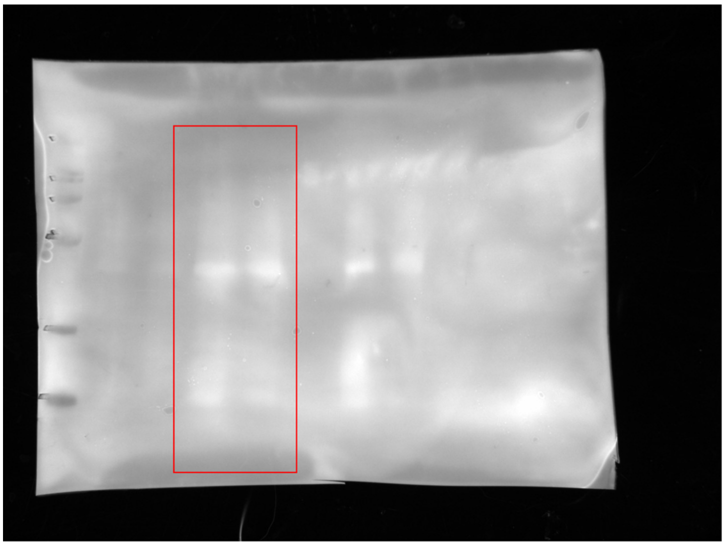

Original images for Fig. 7A (pTorso)

(a)

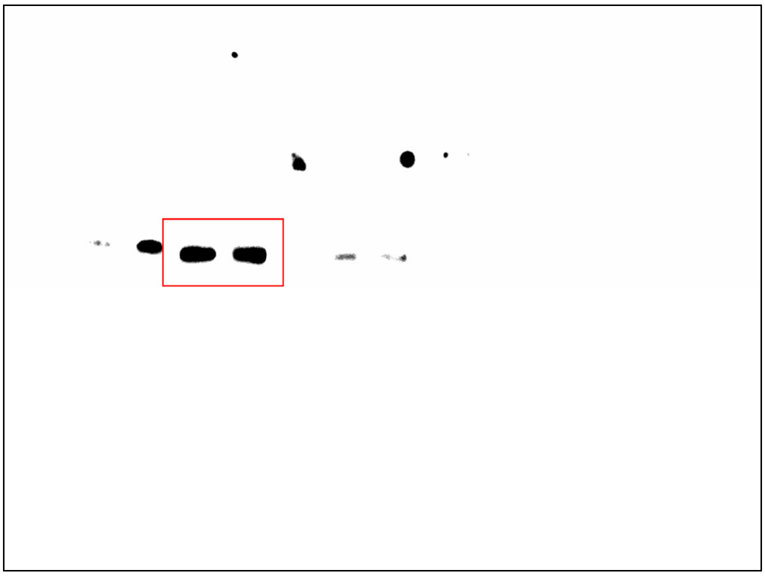

(b)

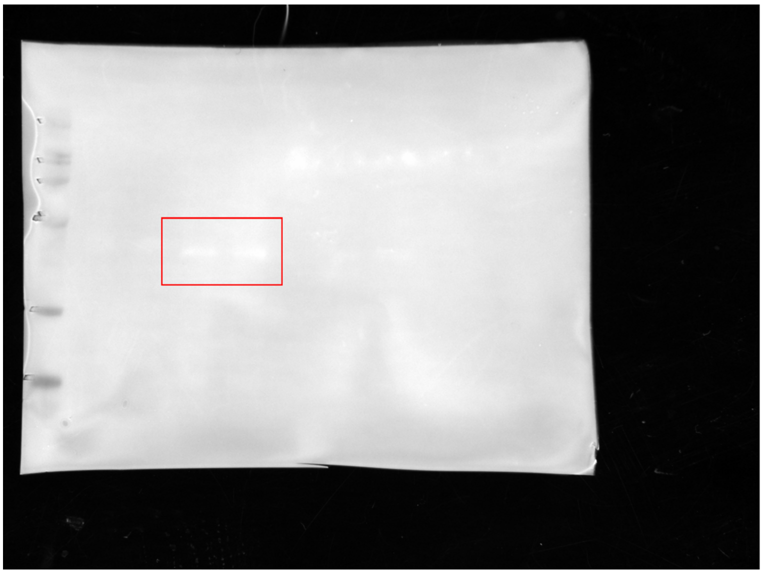

Original images for Fig. 7A (Reprobe)

(a)

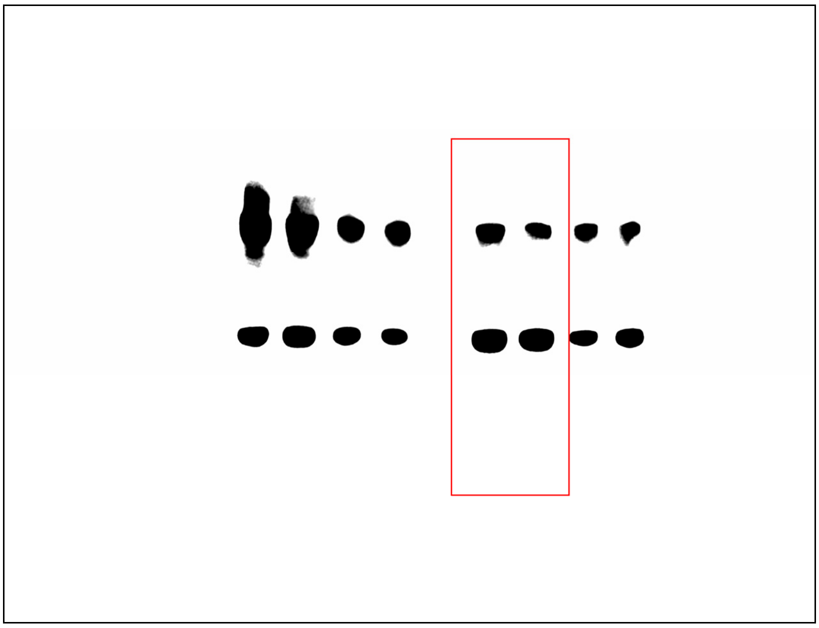

(b)

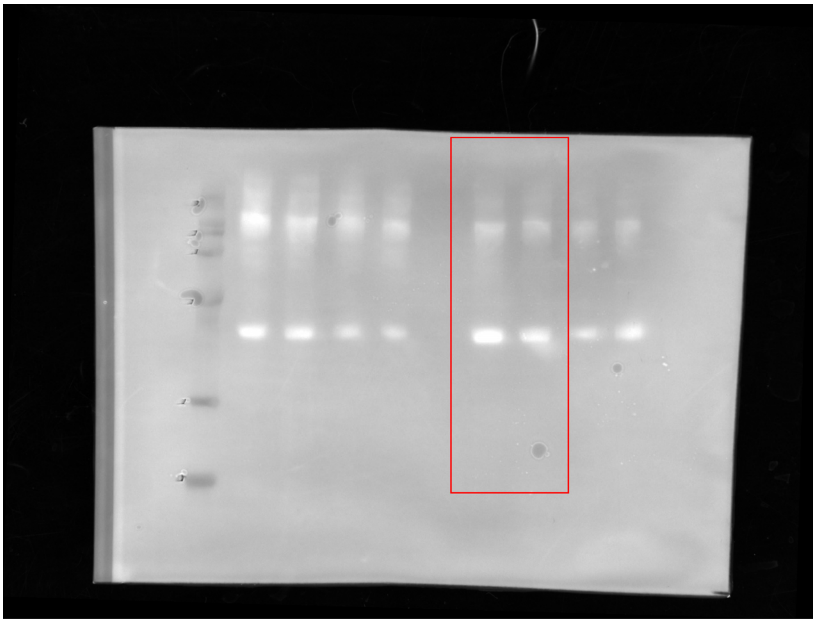

Original images for Fig. 7B

(a)

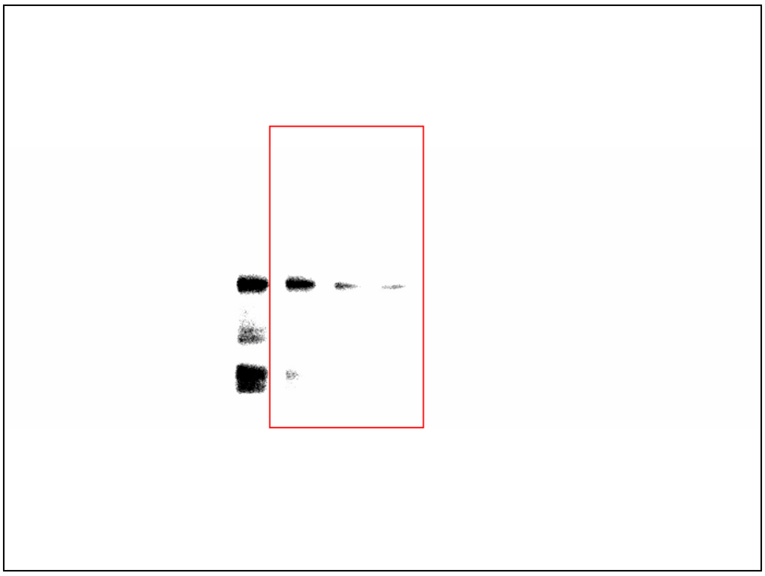

(b)

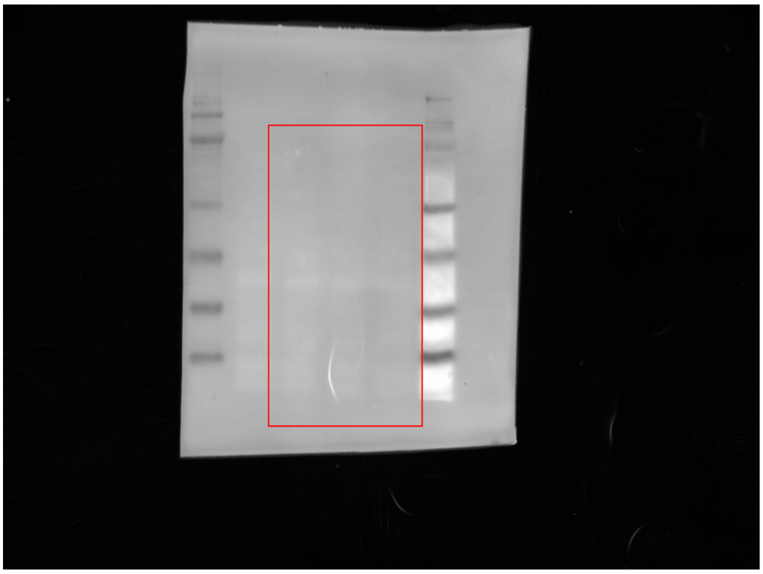

Original images for Fig. 7C (pERK)

(a)

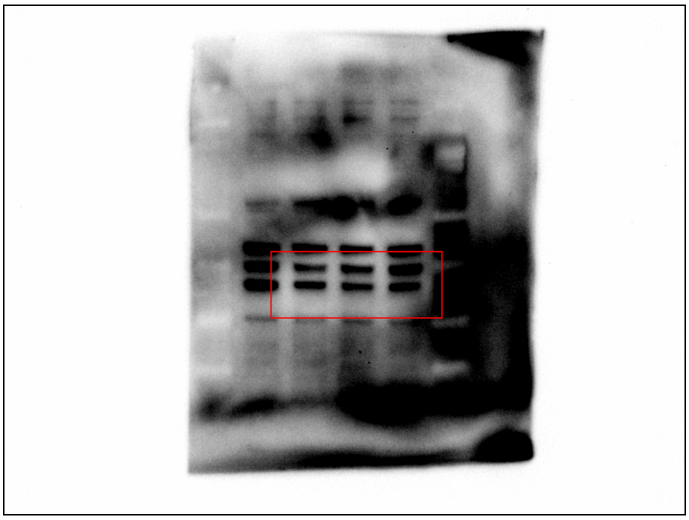

(b)

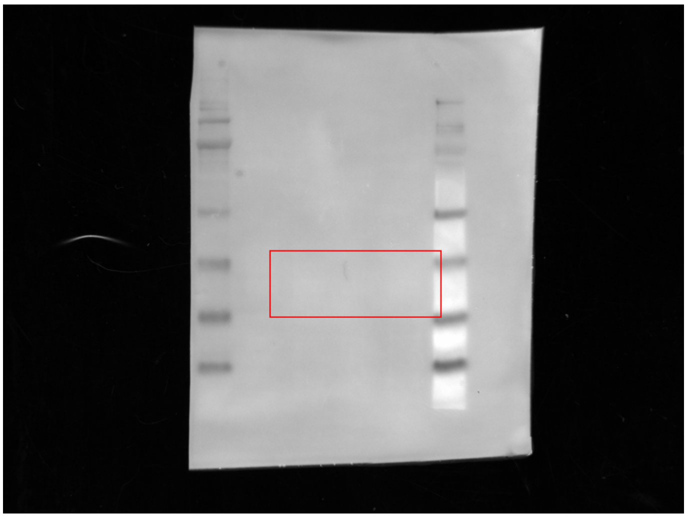

Original images for Fig. 7C (Reprobe)

(a)

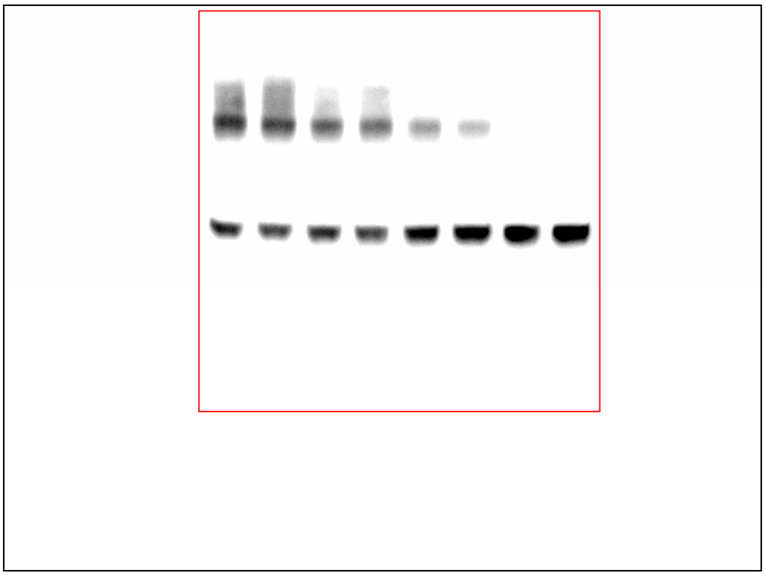

(b)

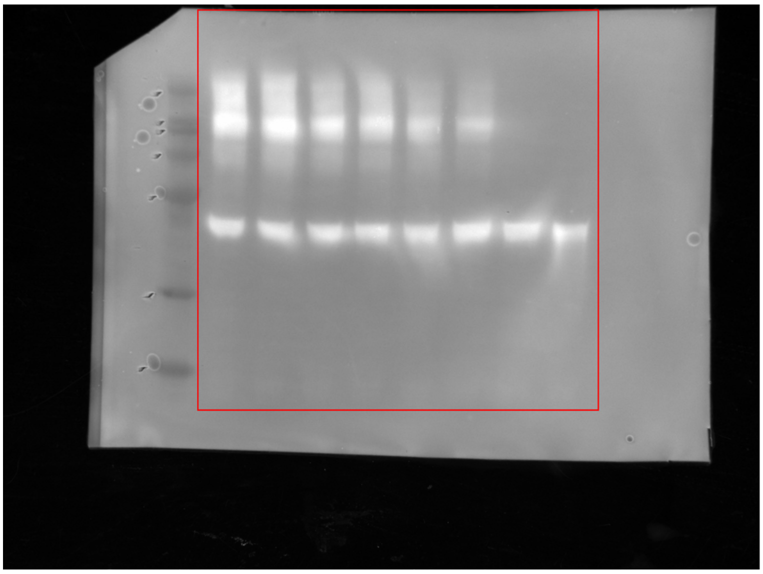

Original images for Fig. S3

(a)

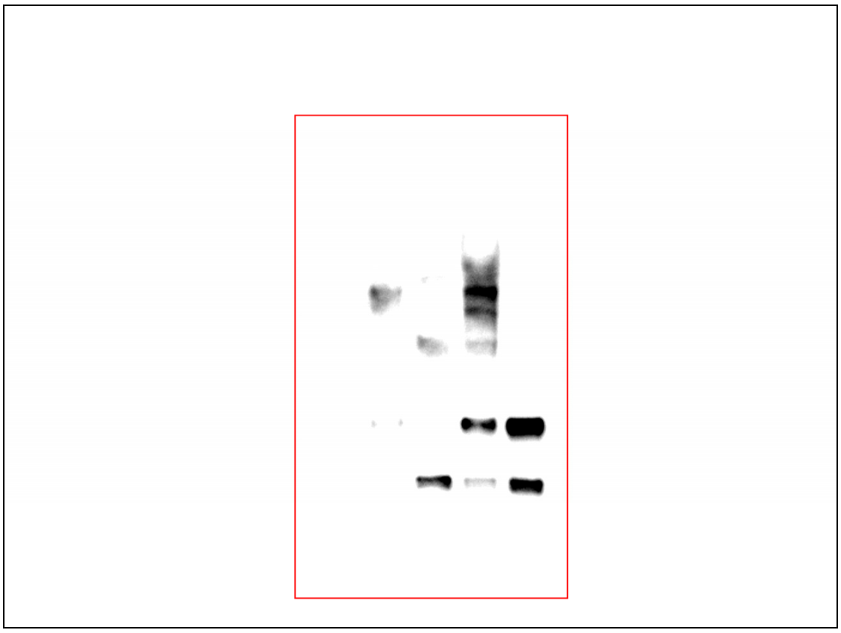

(b)

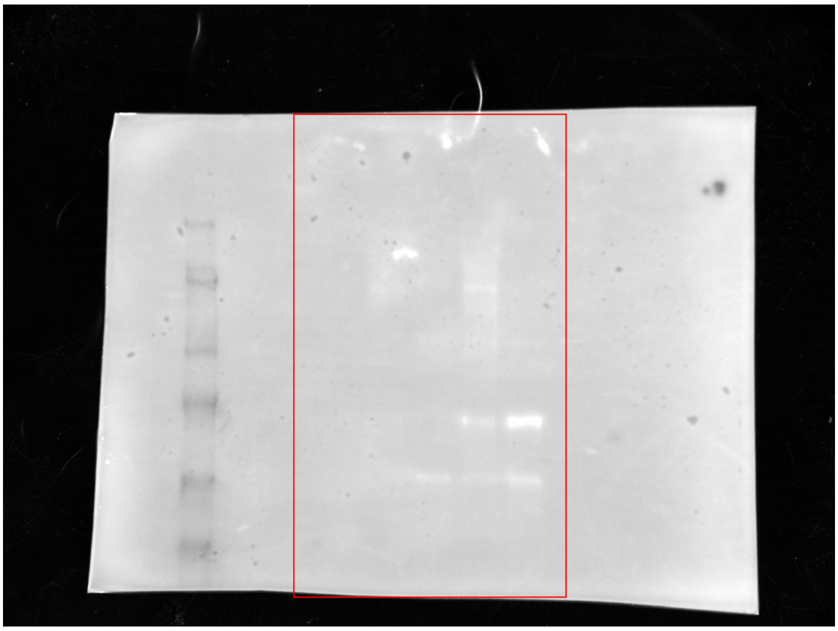

Original images for Fig. S4A

(a)

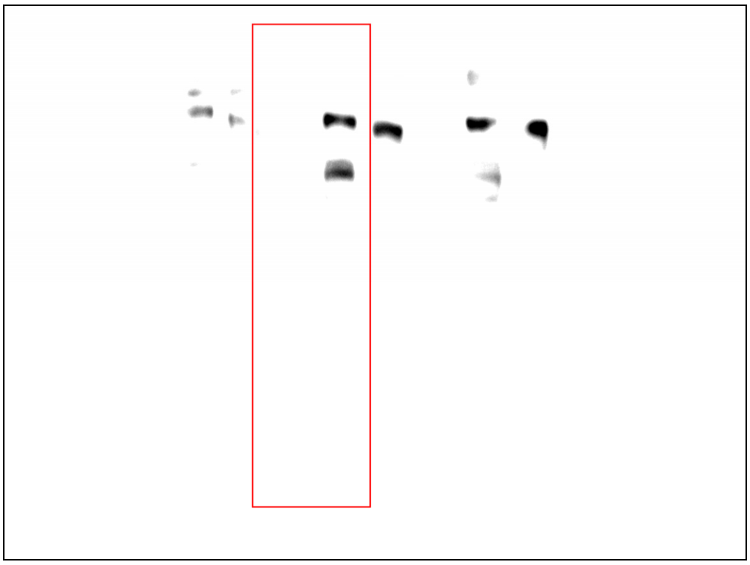

(b)

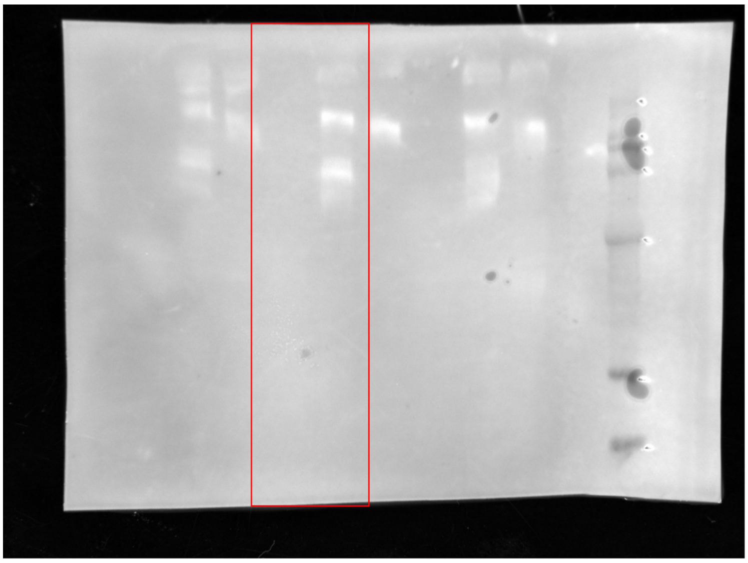

Original images for Fig. S4B

(a)

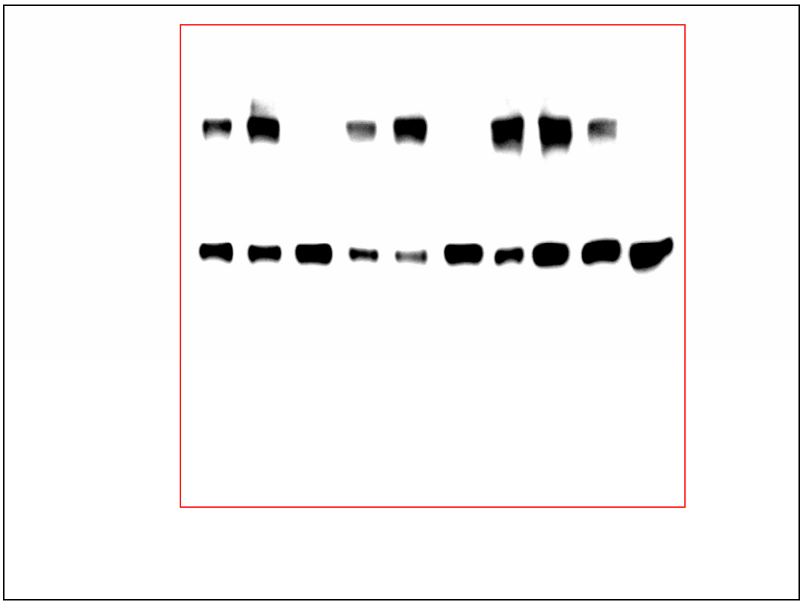

(b)

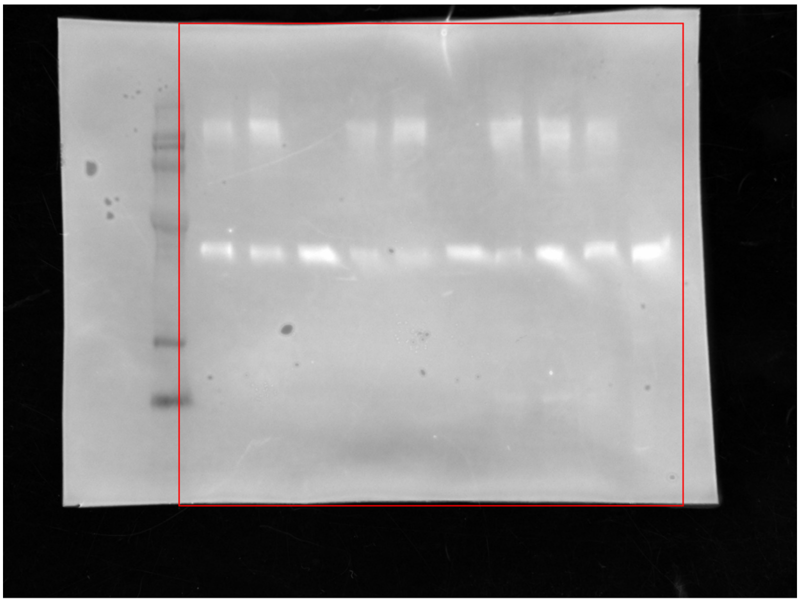

Original images for Fig. S5
